# Biogeography, Assembly Patterns, Driving Factors, and Interactions of Archaeal Community in Mangrove Sediments

**DOI:** 10.1128/mSystems.01381-20

**Published:** 2021-06-15

**Authors:** Zhi-Feng Zhang, Jie Pan, Yue-Ping Pan, Meng Li

**Affiliations:** a Shenzhen Key Laboratory of Marine Microbiome Engineering, Institute for Advanced Study, Shenzhen University, Shenzhen, China; b Key Laboratory of Optoelectronic Devices and Systems of Ministry of Education and Guangdong Province, College of Optoelectronic Engineering, Shenzhen University, Shenzhen, China; University of California San Diego

**Keywords:** archaeal community, neutral community model, beta nearest-taxon index, homogeneous selection, cooccurrence network, mangrove sediment

## Abstract

Archaea are a major part of Earth’s life. They are believed to play important roles in nutrient biogeochemical cycling in the mangrove. However, only a few studies on the archaeal community in mangroves have been reported. In particular, the assembly processes and interaction patterns that impact the archaeal communities in mangroves have not been investigated to date. Here, the biogeography, assembly patterns, and driving factors of archaeal communities in seven representative mangroves across southeastern China were systematically analyzed. The analysis revealed that the archaeal community is more diverse in surface sediments than in subsurface sediments, and more diverse in mangroves at low latitudes than at high latitudes, with *Woesearchaeota* and *Bathyarchaeota* as the most diverse and most abundant phyla, respectively. Beta nearest-taxon index analysis suggested a determinant role of homogeneous selection on the overall archaeon community in all mangroves and in each individual mangrove. In addition, the conditionally rare taxon community was strongly shaped by homogeneous selection, while stochastic processes shaped the dominant taxon and always-rare taxon communities. Further, a moderate effect of environmental selection on the archaeal community was noted, with the smallest effect on the always-rare taxon community. Mangrove location, mean annual temperature, and salinity were the major factors that greatly affected the community composition. Finally, network analysis revealed comprehensive cooccurrence relationships in the archaeal community, with a crucial role of *Bathyarchaeota*. This study expands the understanding of the biogeography, assembly patterns, driving factors, and cooccurrence relationships of the mangrove archaeal community and inspires functional exploration of archaeal resources in mangrove sediments.

**IMPORTANCE** As a key microbial community component with important ecological roles, archaea merit the attention of biologists and ecologists. The mechanisms controlling microbial community diversity, composition, and biogeography are central to microbial ecology but poorly understood. Mangroves are located at the land-ocean interface and are an ideal environment for examining the above questions. We here provided the first-ever overview of archaeal community structure and biogeography in mangroves located along an over-9,000-km coastline of southeastern China. We observed that archaeal diversity in low-latitude mangroves was higher than that in high-latitude mangroves. Furthermore, our data indicated that homogeneous selection strongly controlled the assembly of the overall and conditionally rare taxon communities in mangrove sediments, while the dominant taxon and always-rare taxon communities were mainly controlled by dispersal limitation.

## INTRODUCTION

Mangrove wetlands are widely distributed along the coast in tropical and subtropical regions. They play important ecological roles, such as protecting the coastal area and providing food and shelter for fish and shellfish (1, 2). Although they cover less than 1% of the tropical surface area, they account for 11% of the total input of terrestrial carbon into the ocean and 10% of the terrestrial dissolved organic carbon exported to the ocean, making them a “blue carbon sink” (1, 3, 4). Because of specific ecological features, such as high nutrient concentration and high salinity, low oxygen and low pH, strong redox potential, and vertical sedimentary physicochemical gradients, mangrove ecosystems may harbor numerous adapted organisms (5, 6). Archaea are key components of the microbial community in mangroves, with relative proportions up to 20.8 to 41.3% (7) in the prokaryotic community. They are supposed to have abundant and diverse metabolic pathways (8), such as ammonia oxidation (9, 10), degradation of organic matter (10–12), methane metabolism (13, 14), and sulfate reduction (15, 16), indicating key roles of archaea in driving complex nutrient and biogeochemical cycling in mangrove sediments.

While archaea may have important ecological functions in mangrove and are thus immensely interesting to biologists and ecologists, there is a dearth of studies on archaeal community assembly in mangroves. Yan et al. (17) were the first to reveal a predominance of *Crenarchaeota* and *Euryarchaeota* archaea in mangrove sediments using the analysis of 16S rRNA gene clones. By comparing archaeal communities in pristine and oil-polluted mangroves, Bhattacharyya et al. (18) demonstrated a clear community shift in response to environmental conditions. Recent studies have mainly focused on the effect of spatial, temporal, and environmental variables on the archaeal community. For instance, seasonality, sediment depth, and pH have important effects on the archaeal community (7, 19–22), and total organic carbon (TOC) and nitric oxide are significantly correlated with the abundance of *Bathyarchaeota* (22). However, most of the above studies of the archaeal community were focused on individual mangroves and seldom provide an integrative view of the influence patterns of spatial and environmental parameters on a large geographical scale.

Understanding the forces that mold the community composition structure is a major goal of microbial ecology (23). Microbial community assembly and the mechanisms shaping the community diversity, distribution, and biogeography are central but poorly understood topics of aquatic microbial ecology (23–26). Community assembly is simultaneously shaped by deterministic and stochastic factors (23, 25, 26). The former include selection imposed by abiotic (environmental factors) and biotic (species interactions) factors, and the latter include unpredictable ecological events, such as birth, death, immigration, speciation, and limited dispersal (23, 25). As fluctuating ecosystems at the land-ocean interface with important ecological roles, mangroves provide a unique environment wherein to examine the community assembly theories. Although there are several studies exploring microbial community composition and the influencing factors in mangroves, few have tried to dissect the relative importance of stochastic and deterministic processes therein. Zhang et al. (13) reported a relatively more important role of the deterministic process rather than of the stochastic process in shaping the entire prokaryotic community assembly in mangroves. However, the knowledge of archaeal community assembly in mangroves is still limited.

According to several studies, some taxa with low relative abundance (i.e., rare taxa) are in fact metabolically active and may act as keystones that regulate the functions of aquatic ecosystems. Hence, the microbes of the “rare biosphere” have important roles in the metabolic and ecological functions of aquatic habitats (26–28). For example, rare archaea are supposed to have ecological functions, such as acetate metabolism (29), carbon cycling (30), and methanogenesis (31). Another interesting component is the taxa which are usually rare but occasionally become more prominent under optimal conditions, namely, the conditionally rare taxa (26, 28, 32). Conditionally rare taxa can explain large temporal shifts of microbial structure (29, 33). However, the knowledge of many ecosystems, including mangrove wetlands, is mostly based on dominant or entire communities, with the roles of always-rare taxa and conditionally rare taxa rarely accounted for (29). Consequently, to explore the assembly patterns, biogeography, and potential controlling factors of the “rare biosphere” in mangrove sediments, the archaeal community in the current study was separated into dominant taxa, conditionally rare taxa, and always-rare taxa.

Accordingly, in the current study, we aimed to shed light on the processes that govern the archaeal community assembly in mangroves on a large scale. Large-scale sampling could provide data for estimating natural distributions and building species distribution models (34). To do this, we characterized the archaeal communities in 127 samples from seven representative mangroves along the Southeast China coast (see Data Set S1, sheet 1, in the supplemental material). We first explored the diversity, biogeography, and composition of archaeal communities. Then, we quantified the community assembly processes using neutral community model (NCM) fitting and beta nearest-taxon index (βNTI). In addition, we determined the selection by environmental factors using variation partition analysis (VPA) and distance-based redundancy analysis (db-RDA). Finally, we explored species interactions within the archaeal community based on cooccurrence correlation. The analysis allowed us to address the following questions. (i) Do all, dominant, conditionally rare, and always-rare archaeal taxa exhibit similar or different biogeography and community compositions? (ii) Do all, dominant, conditionally rare, and always-rare archaeal taxa assemble via different community assembly processes? (iii) How much do the deterministic and stochastic processes control the community assembly? (iv) Which environmental factor has the greatest effect on archaeal community composition?

10.1128/mSystems.01381-20.9 DATA SET S1 Sheet 1, sample information and α-diversity. Sheet 2, OTU annotation and distribution in different samples. Sheet 3, Keystone taxa in the overall network with the highest keystoneness scores. Download Data Set S1, XLSX file, 9.1 MB. Copyright © 2021 Zhang et al. 2021 Zhang et al. https://creativecommons.org/licenses/by/4.0/ This content is distributed under the terms of the Creative Commons Attribution 4.0 International license.

## RESULTS

### Composition and diversity of archaeal community in mangrove sediments.

In the study, seven representative mangroves (DZG, FT, SK, XMD, YLW, ZJ, and ZJK) along the Southeast China coast were sampled (127 samples; see Data Set S1, sheet 1, in the supplemental material). Then, 16S rRNA genes were sequenced from samples from different sediment depths. After quality filtering and chimeric sequence removal, 4,926,673 high-quality sequences were clustered into 23,481 operational taxonomic units (OTUs), of which 15,514 were assigned as *Archaea*. After bacterial OTU removal, the archaeal OTU table was used for the ensuing analyses. Between 19,206 and 43,750 archaeal sequences were detected in each sample, with an average of 33,963 sequences per sample (Data Set S1, sheet 2). Rarefaction curve analysis indicated that most samples reached saturation (Fig. S1a), and archaeal OTU accumulation curves for different mangroves were nearly asymptotic (Fig. S1b). Comparison of α-diversity indices revealed higher archaeal richness in the 0- to 10-cm depth layer than that in the 20- to 30-cm layer in terms of OTU richness (*P* < 0.05), while none of the other indices were significantly different among the different depth layers (Fig. S2).

10.1128/mSystems.01381-20.1 FIG S1 Rarefaction curves for each sample (a) and different mangroves (b). Download FIG S1, JPG file, 2.0 MB. Copyright © 2021 Zhang et al. 2021 Zhang et al. https://creativecommons.org/licenses/by/4.0/ This content is distributed under the terms of the Creative Commons Attribution 4.0 International license.

10.1128/mSystems.01381-20.2 FIG S2 Comparisons of operational taxonomic unit (OTU) richness (a), Shannon (b), Chao1 (c), and evenness indices (d) at different sediment depths, as demonstrated by boxplots, with the median and 95% confidence interval displayed. The *P* values are denoted for each comparison: ns, not significant; *, *P* ≤ 0.05; **, *P* ≤ 0.01; ***, *P* ≤ 0.001. Download FIG S2, JPG file, 0.8 MB. Copyright © 2021 Zhang et al. 2021 Zhang et al. https://creativecommons.org/licenses/by/4.0/ This content is distributed under the terms of the Creative Commons Attribution 4.0 International license.

**FIG 1:**
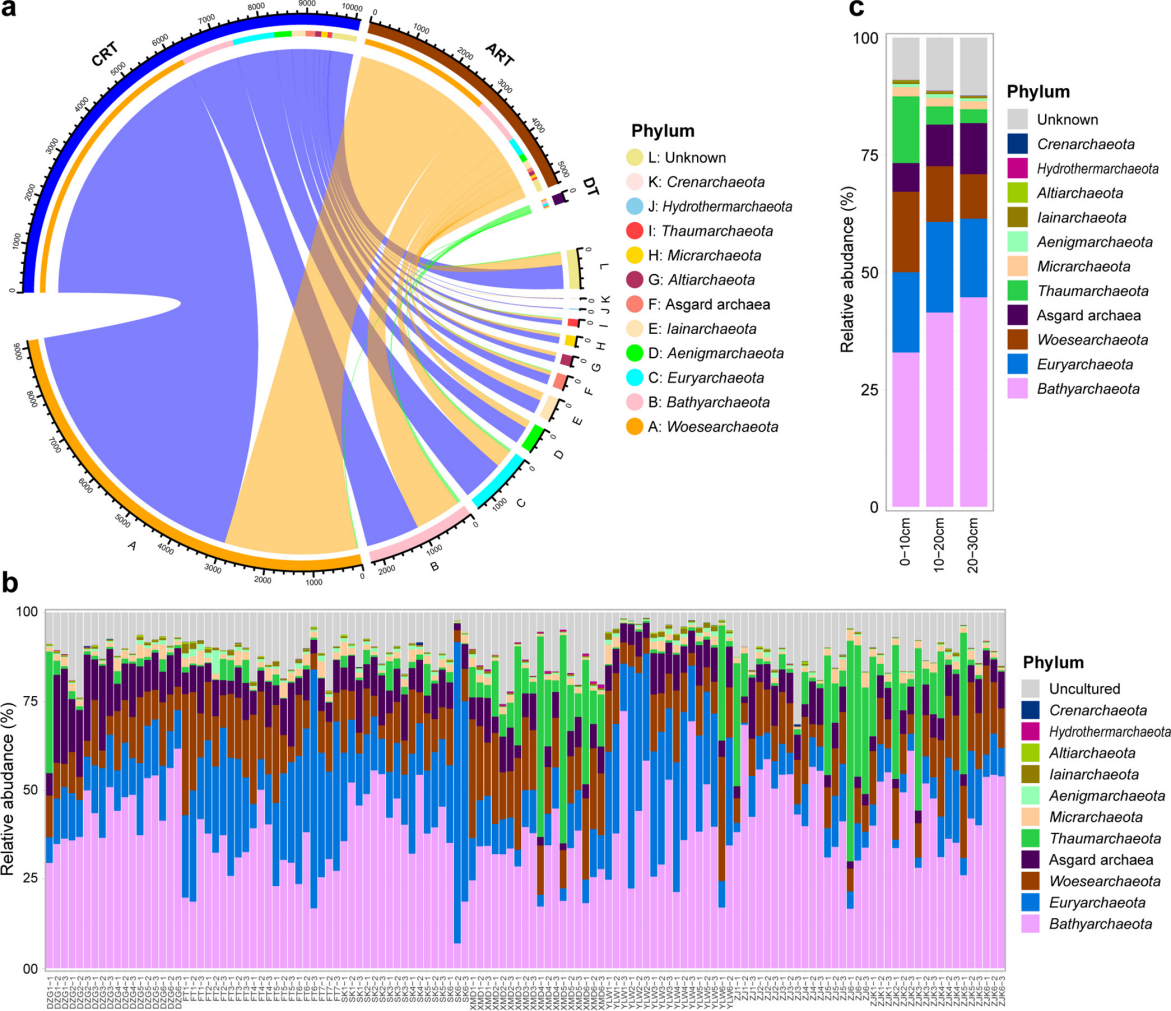
Taxonomic composition of the archaeal community. (a) Archaeal OTUs of dominant taxa (DT), conditionally rare taxa (CRT), and always-rare taxa (ART) at phylum level. The colors of the top half of the outer ring represent DT, CRT, and ART, respectively. The lower half of the outer ring is colored according to the phyla specified in the key. Line thickness corresponds to the numbers of OTUs in different groups. (b) Relative abundance of phyla in each sample. (c) Relative abundance of phyla in three depth layers.

Although *Woesearchaeota* exhibited the highest OTU richness (58.9% of OTUs, 12.8% of archaeal sequences), *Bathyarchaeota* was the most abundant archaeal phylum in most samples, accounting for 7.0% (SK6-2) to 72.3% (ZJ1-2) of archaeal sequences (Fig. 1a and b; Data Set S1, sheet 2), with an overall sequence proportion of 39.8%. *Euryarchaeota* (8.8% of OTUs, 17.8% of sequences) and Asgard archaea (2.0% of OTUs, 8.7% of sequences) were also predominant in the mangrove archaeal community. The relative abundance of Asgard archaea and *Bathyarchaeota* in the 10- to 20-cm and 20- to 30-cm depth layers was significantly higher than that in the 0- to 10-cm depth layer (*P* < 0.05). The relative abundance of *Thaumarchaeota* and *Woesearchaeota* was significantly higher in the 0- to 10-cm depth layer than in the 10- to 20-cm and 20- to 30 -cm depth layers (*P* < 0.015) (Fig. S3).

10.1128/mSystems.01381-20.3 FIG S3 Comparisons of the main archaeal phyla and classes at different sediment depths, as demonstrated by boxplots, with the median and 95% confidence interval displayed. The *P* values are denoted for each comparison: ns, not significant; *, *P* ≤ 0.05; **, *P* ≤ 0.01; ***, *P* ≤ 0.001; ****, *P* ≤ 0.0001. Download FIG S3, JPG file, 1.9 MB. Copyright © 2021 Zhang et al. 2021 Zhang et al. https://creativecommons.org/licenses/by/4.0/ This content is distributed under the terms of the Creative Commons Attribution 4.0 International license.

According to the criteria described in Materials and Methods, a high proportion of OTUs were identified as always-rare taxa (mean = 33.6%) and conditionally rare taxa (mean = 65.1%), but they accounted for only 0.7% and 31.0% of the average relative abundance in each sample, respectively. Conversely, a low proportion of OTUs (mean = 1.3%) were identified as dominant taxa and accounted for 68.3% of the average relative abundance in each sample (Fig. 1a; Data Set S1, sheet 2).

**FIG 2:**
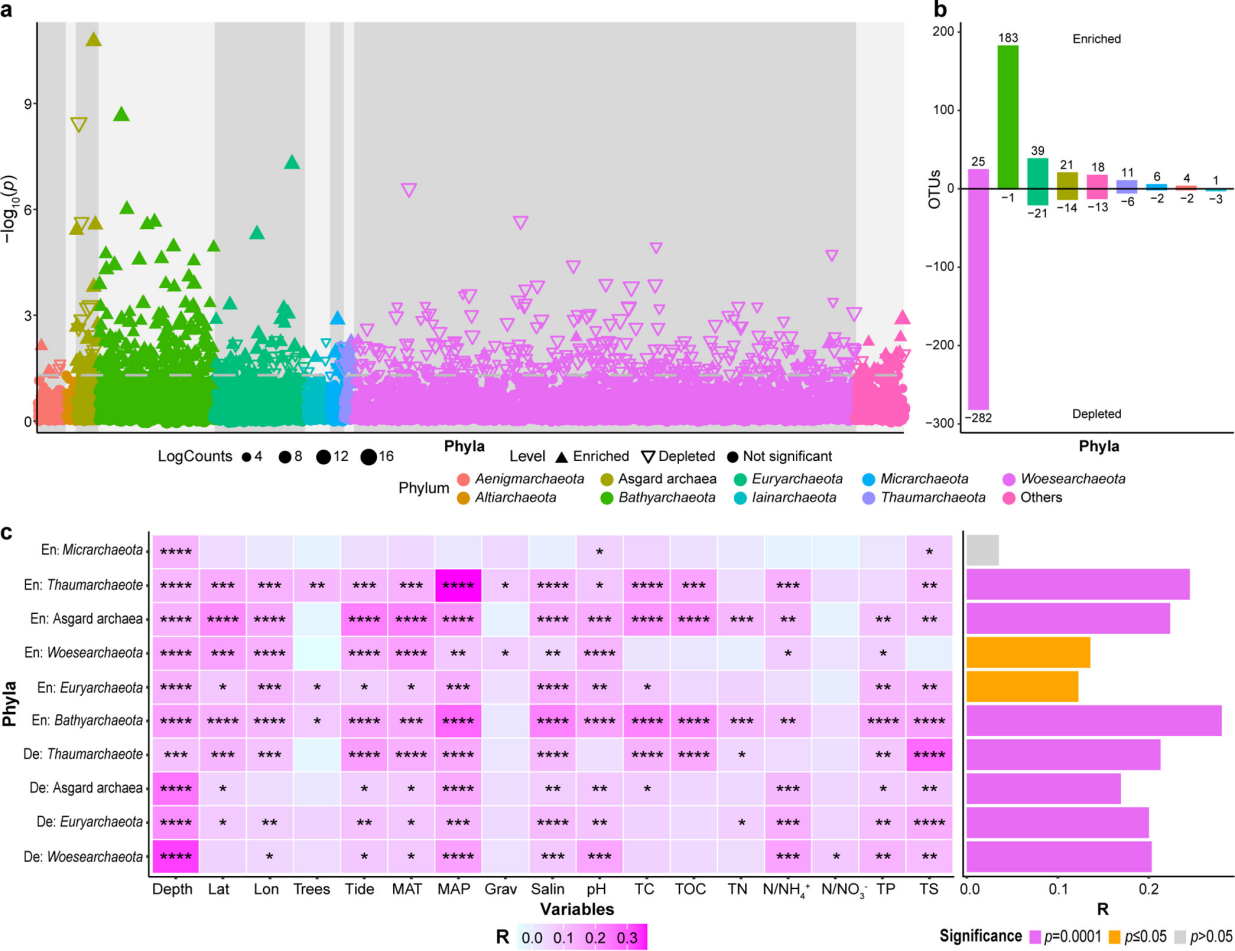
Comparison of OTUs’ distribution between surface and subsurface mangrove sediments. (a) Comparison of OTU composition in the surface (0- to 10-cm) sediment samples and subsurface (10- to 30-cm) sediment samples in mangroves. Counts, total read number. Dashed line, 95% confidence level. Solid upward triangles, OTUs significantly enriched in subsurface sediments. Hollow downward triangles, OTUs significantly depleted in subsurface sediments. Solid points, OTUs with unchanged abundance in subsurface sediments compared with surface sediments. Triangle and point sizes are proportional to the abundance of that OTU. (b) Numbers of enriched and depleted OTUs in subsurface sediments compared with those in surface sediments at phylum level. (c) Mantel test for the effects of environmental variables on enriched and depleted phyla in subsurface sediments. Left, the effects of individual variables, denoted by the color gradient: *, *P* ≤ 0.05; **, *P* ≤ 0.01; ***, *P* ≤ 0.001; ****, *P* ≤ 0.0001. Right, the effects of all variables combined.

Comparison of the archaeal OTUs in the subsurface (10- to 30-cm) and surface (0- to 10-cm) sediments revealed a higher count of depleted OTUs (344 OTUs, 2.2% of archaeal OTUs) than enriched OTUs (308 OTUs, 1.9% of archaeal OTUs) in the subsurface sediment layers (Fig. 2a and b). Most of the enriched OTUs belonged to *Bathyarchaeota* (183 OTUs, 59.4% of enriched OTUs), *Euryarchaeota* (39 OTUs, 12.7% of enriched OTUs), *Woesearchaeota* (25 OTUs, 8.1% of enriched OTUs), Asgard archaea (21 OTUs, 6.8% of enriched OTUs), *Thaumarchaeota* (11 OTUs, 3.6% of enriched OTUs), and *Micrarchaeota* (6 OTUs, 2.0% of enriched OTUs). The depleted OTUs mainly belonged to *Woesearchaeota* (282 OTUs, 82.0% of depleted OTUs), *Euryarchaeota* (21 OTUs, 6.1% of depleted OTUs), Asgard archaea (14 OTUs, 4.1% of depleted OTUs), and *Thaumarchaeota* (6 OTUs, 1.7% of depleted OTUs). This suggested pronounced depletion of *Woesearchaeota* and enrichment of *Bathyarchaeota* in deeper sediments, which exhibited higher and lower relative abundance in the 10- to 30-cm depth layers than in the 0- to 10-cm depth layer, respectively (Fig. S3). Depleted *Woesearchaeota* OTUs were significantly affected by depth, longitude, tidal height, mean annual temperature (MAT), mean annual precipitation (MAP), salinity, pH, ammonium nitrogen (N/NH_4_^+^), nitrate nitrogen (N/NO_3_^−^), total phosphorus (TP), and total sulfur (TS) (Mantel test, *P < *0.05), and the correlation coefficient for all variables combined was 0.2030 (Mantel test, *P* = 0.0001) (Fig. 2c). In contrast, the enriched *Woesearchaeota* OTUs were further significantly affected by the latitude and gravel proportion, instead of N/NO_3_^−^ and TS (Mantel test, *P* < 0.05), and the correlation with all variables combined was 0.1356 (Mantel test, *P* = 0.0001) (Fig. 2c). The enriched *Bathyarchaeota* OTUs were significantly influenced by almost all variables evaluated in the study, individually (Mantel test, *P* < 0.05) and combined (Mantel test, *R* = 0.2800, *P* = 0.0001) (Fig. 2c). Further, the remaining phyla with more than five depleted or enriched OTUs, except for the depleted *Micrarchaeota*, were significantly affected by all variables combined (Mantel test, *P* < 0.05) (Fig. 2c).

### Archaeal community assembly patterns in mangrove ecosystems.

**FIG 3:**
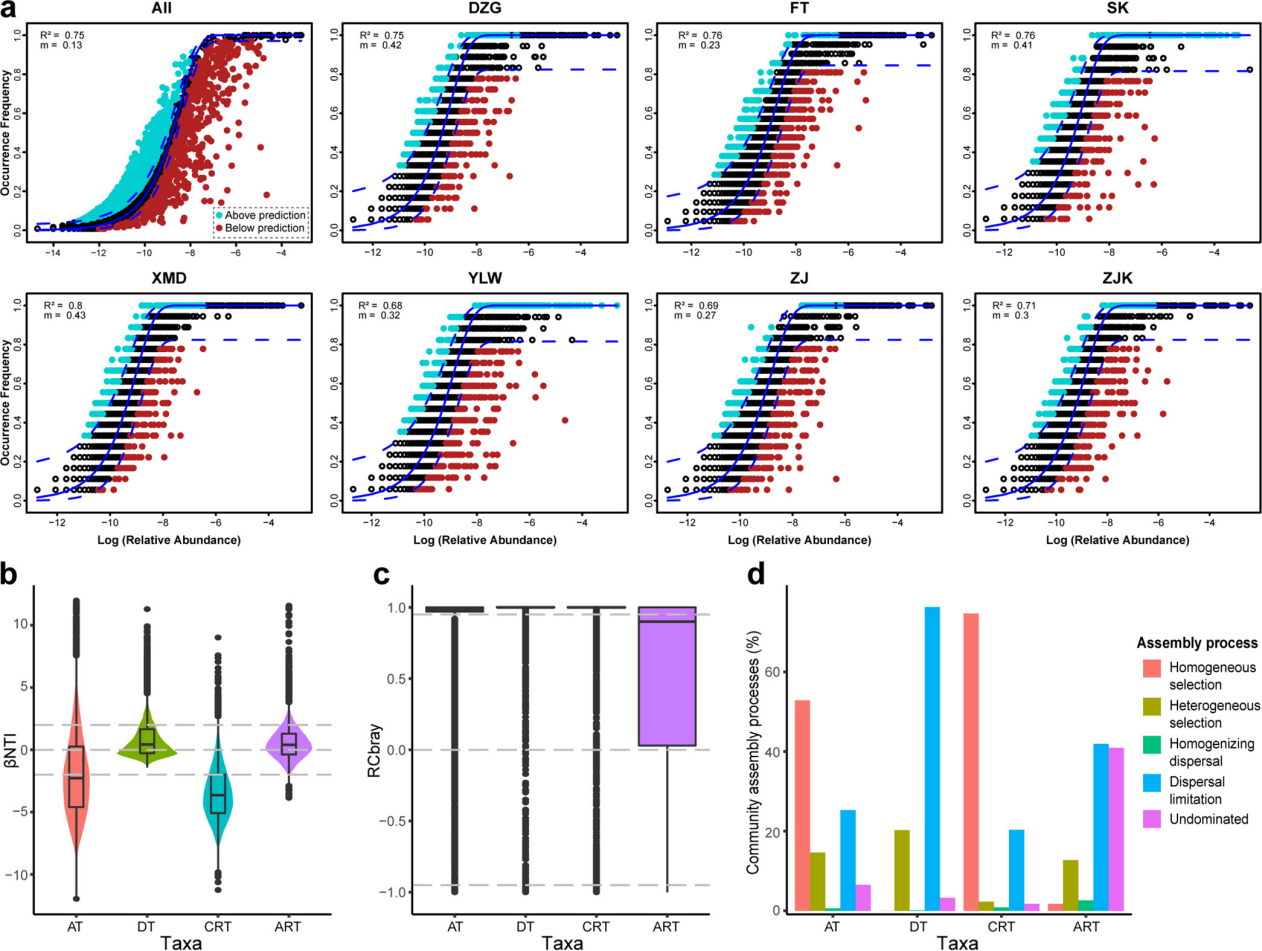
Archaeal community assembly patterns. (a) Fitting of the neutral community model (NCM) of community assembly in all and individual mangroves. Solid blue lines, the best fit to NCM. Dashed blue lines, 95% confidence interval for the predication. OTUs that occur more or less frequently than predicted by the NCM are denoted by different colors. *m*, immigration rate; *R*^2^, fit to the model. (b) β-nearest-taxon index (βNTI) of all-taxon (AT), dominant taxon (DT), conditionally rare taxon (CRT), and always-rare taxon (ART) communities in all mangroves. Horizontal dashed lines (βNTI values at 2 and −2), thresholds of significance. (c) Bray-Curtis-based Raup-Crick (RC_bray_) values of AT, DT, CRT, and ART communities in all mangroves. Horizontal dashed lines, RC_bray_ values at 0.95 and −0.95. (d) The percent turnover of AT, DT, CRT, and ART community assembly governed primarily by various deterministic processes, including homogenous and heterogeneous selections, and stochastic processes, including dispersal limitations and homogenizing dispersal, as well as “Undominated” processes.

The NCM fitted well the archaeal community assembly in all and individual mangroves (*R*^2^ > 0.6) (Fig. 3a). The estimated immigration rate (*m*) was much higher in individual mangroves (0.23 to 0.43) than that in all mangroves (Fig. 3a), suggesting the occurrence of more dispersal events and ecological drift within each mangrove than among mangroves. To further explore the relative contribution of the stochastic and deterministic processes to the archaeal community assembly, βNTI and Bray-Curtis-based Raup-Crick (RC_bray_) were calculated based on the OTU abundance and their phylogenetic distance. For all taxa, while the average βNTI value (−1.92) in all mangroves was slightly higher than −2, the threshold of stochastic and deterministic processes, the majority of βNTI values (67.5%) in all mangroves were lower than −2 or higher than 2 (Fig. 3b). This indicated that the deterministic processes are more important for community assembly than the stochastic processes. Further, the majority (78.0%) of RC_bray_ values were greater than 0.95, indicating a more important role of dispersal limitation in the community assembly than that of homogenizing dispersal and “Undominated” processes (Fig. 3c). Consequently, based on the criteria described in Materials and Methods, all taxon community assembly patterns in mangroves were assigned to five portions: homogeneous selection, heterogeneous selection, homogenizing dispersal, dispersal limitation, and “Undominated” processes. Of these, homogeneous selection (52.9%), followed by dispersal limitation (25.3%), was the most crucial process controlling the community assembly (Fig. 3d). Similarly, homogeneous selection played a crucial role in the conditionally rare taxon community assembly (Fig. 3b to d). In contrast, the dominant assembly process controlling the dominant taxon (76.3%) and always-rare taxon (41.9%) community assembly was dispersal limitation. The roles of “Undominated” processes (41.0%) in the always-rare taxon community assembly were almost equal to those of dispersal limitation (Fig. 3d). For each mangrove sampled in the current study, homogeneous selection was the dominant assembly process (38.2 to 64.7%), followed by dispersal limitation (19.7 to 33.5%) (Fig. S4a to c).

10.1128/mSystems.01381-20.4 FIG S4 (a) β-nearest-taxon index (βNTI) of archaeal community in individual mangroves. Horizontal dashed lines (βNTI values at 2 and −2), thresholds of significance. (b) Bray-Curtis-based Raup-Crick (RC_bray_) values of archaeal community in individual mangroves. Horizontal dashed lines, RC_bray_ values at 0.95 and −0.95. (c) The percent turnover of archaeal community assembly governed primarily by various deterministic processes, including homogenous and heterogeneous selections, and stochastic processes, including dispersal limitations and homogenizing dispersal, as well as “Undominated” processes. (d) The relationships between βNTI and changes in the tidal height (Tide), mean annual temperature (MAT), mean annual precipitation (MAP), gravel proportion, salinity, pH, total carbon (TC), total organic carbon (TOC), total nitrogen (TN), N/MH_4_^+^, N/NO_3_^−^, total phosphorus (TP), and total sulfur (TS). Statistical significance is displayed as follows: *, *P* ≤ 0.05; **, *P* ≤ 0.01; ***, *P* ≤ 0.001; ****, *P* ≤ 0.0001. Download FIG S4, JPG file, 1.5 MB. Copyright © 2021 Zhang et al. 2021 Zhang et al. https://creativecommons.org/licenses/by/4.0/ This content is distributed under the terms of the Creative Commons Attribution 4.0 International license.

The correlations between βNTI values and changes in the environmental variables were further explored (Fig. S4d). βNTI values were significantly correlated with the changes of several variables, especially for the conditionally rare taxon community, for which the βNTI values were significantly correlated with all variables except for tidal height. Among the variables, changes in MAP and salinity were most strongly correlated with βNTI (Fig. S4d). However, all correlations were quite weak (|*R *|< 0.22) (Fig. S4d). Further, Bray-Curtis similarity values of archaeal communities (including all-taxon, always-rare taxon, conditionally rare taxon, and dominant taxon communities) in all samples and different depth layers were significantly and negatively correlated with the geographical distance, indicating a significant distance-decay relationship between them (*P* < 0.001, Fig. S5). In addition, Bray-Curtis similarity of the dominant taxon communities was significantly higher than that of the conditionally rare taxon and always-rare taxon communities, and that of the conditionally rare taxon communities was also significantly higher than that of the always-rare taxon communities, suggesting a stronger environmental preference or limitation of ecological dispersal of always-rare taxa than that of conditionally rare taxa and dominant taxa (Fig. S6).

10.1128/mSystems.01381-20.5 FIG S5 Distance-decay patterns of archaeal community similarity based on the geographic distance and Bray-Curtis similarity of archaeal community compositions of all taxa, dominant taxa, conditionally rare taxa, and always-rare taxa in all samples (a) and in samples at different depth layers (b). The shaded area around the lines covers the 95% confidence interval of the correlations. The associated correlation coefficients and *P* values are shown in each panel. Download FIG S5, JPG file, 2.5 MB. Copyright © 2021 Zhang et al. 2021 Zhang et al. https://creativecommons.org/licenses/by/4.0/ This content is distributed under the terms of the Creative Commons Attribution 4.0 International license.

10.1128/mSystems.01381-20.6 FIG S6 Comparisons of the similarities of archaeal community compositions in all samples and in samples at different depth layers. The *P* values are shown for all comparisons. Download FIG S6, JPG file, 0.8 MB. Copyright © 2021 Zhang et al. 2021 Zhang et al. https://creativecommons.org/licenses/by/4.0/ This content is distributed under the terms of the Creative Commons Attribution 4.0 International license.

### Spatial and environmental selection shaping the archaeal community composition.

Regarding the α-diversity indices, OTU richness was positively correlated with MAT, gravel proportion, pH, total nitrogen (TN), N/NO_3_^−^, and TS, but it was negatively correlated with latitude, longitude, depth, tidal height, and TP. Shannon index was positively correlated with gravel proportion, pH, N/NO_3_^−^, and TS but negatively correlated with N/NH_4_^+^ and TP (Table 1). Correlation analyses were further performed to explore the relationships between the environmental variables and the relative abundance of main phyla and classes (Table 1). The relative abundance of *Bathyarchaeota* was significantly positively correlated with sediment depth, MAT, and TS but negatively correlated with latitude, longitude, tidal height, pH, and N/NH_4_^+^. The relative abundance of *Woesearchaeota* was negatively correlated with depth, MAT, pH, and TS and positively correlated with latitude, longitude, and tidal height. Furthermore, the relative abundance of *Euryarchaeota* was significantly correlated with all variables except for depth, MAP, salinity, and total carbon (TC), among which it was negatively correlated with latitude, longitude, and tidal height. Depth, MAT, TC, TOC, and TS were significantly positively correlated with the relative abundance of Asgard archaea, while latitude, longitude, tidal height, and TP showed negative correlations (Table 1). The above observations indicated significantly lower archaeal richness in high-latitude mangroves than in low-latitude mangroves. Similarly, compared with low-latitude mangroves, the relative abundance of Asgard archaea, *Bathyarchaeota*, and *Euryarchaeota* was significantly reduced in high-latitude mangroves. However, the relative abundance of *Thaumarchaeota* and *Woesearchaeota* was significantly higher at high latitudes than at low latitudes.

**TABLE 1:** Correlation coefficients (Spearman’s rho) for the OTU richness, α-diversity, and relative abundance of main phyla and classes with environmental factors

Parameter/phylum	Correlation coefficient (ρ)^*a*^															
	Latitude	Longitude	Depth	Tide	MAT	MAP	Gravel	Salinity	pH	TC	TOC	TN	N/NH_4_^+^	N/NO_3_^−^	TP	TS
OTU richness	−0.209**	−0.199*	−0.309***	−0.191*	0.219**	−0.108	0.239**	0.055	0.150*	0.128	0.121	0.211**	−0.042	0.218**	−0.180*	0.189*
Shannon-Wiener	−0.028	−0.008	−0.110	−0.030	0.043	−0.085	0.150*	0.055	0.148*	0.071	0.060	0.138	−0.199*	0.172*	−0.267**	0.156*
Chao1	−0.334****	−0.362****	−0.0410	−0.318***	0.316***	0.085	0.149*	−0.127	0.112	0.116	0.128	0.211**	0.065	−0.038	−0.060	0.203*
Evenness	0.026	0.0503	−0.040	0.017	−0.011	−0.072	0.112	0.044	0.131	0.053	0.042	0.110	−0.234**	0.150*	−0.280***	0.133
All Asgard phyla	−0.231**	−0.221**	0.423****	−0.253**	0.231**	0.064	0.018	−0.027	−0.043	0.152*	0.179*	0.133	−0.141	0.116	−0.302***	0.339****
*Bathyarchaeota*	−0.253**	−0.249**	0.375****	−0.211**	0.237**	−0.086	0.032	−0.129	−0.181*	0.089	0.073	0.063	−0.316***	−0.007	−0.397****	0.204*
*Euryarchaeota*	−0.322***	−0.285***	0.018	−0.360****	0.374****	−0.016	0.326****	0.123	0.454****	0.184	0.217**	0.157*	0.626****	0.183*	0.322***	0.300***
*Lokiarchaeota*	−0.287***	−0.266**	0.407****	−0.321***	0.286***	0.113	0.025	−0.096	−0.090	0.188*	0.218**	0.150*	−0.125	0.092	−0.306***	0.362****
*Micrarchaeota*	−0.111	−0.099	−0.068	−0.172*	0.121	0.229**	0.014	−0.283***	0.029	0.083	0.082	0.161*	0.133	0.018	0.103	0.055
*Odinarchaeota*	−0.182*	−0.208**	0.103	−0.136	0.205*	−0.223**	0.101	0.187*	0.144	0.001	0.024	0.154*	−0.018	0.207**	−0.185*	0.171*
*Thaumarchaeota*	0.310***	0.289***	−0.376****	0.362****	−0.345****	−0.101	−0.243**	0.083	−0.139	−0.256**	−0.255**	−0.183*	−0.159*	−0.076	0.096	−0.440****
*Woesearchaeota*	0.356****	0.390****	−0.513****	0.296***	−0.335****	0.084	−0.087	−0.001	−0.204*	−0.044	−0.092	0.024	−0.028	−0.076	0.104	−0.249**
*Methanomicrobia*	−0.130	−0.062	−0.166*	−0.245**	0.164*	0.307***	0.130	−0.257**	0.165*	0.261**	0.261**	0.150*	0.489****	−0.053	0.331****	0.231**
*Methanosarcinia*	−0.110	−0.149*	−0.009	−0.088	0.129	−0.082	0.074	0.180*	0.275***	−0.031	−0.025	−0.049	0.593****	0.016	0.485****	0.008
*Micrarchaeia*	−0.111	−0.099	−0.068	−0.172*	0.121	0.229**	0.014	−0.283***	0.029	0.083	0.082	0.161*	0.133	0.018	0.103	0.055
*Nitrososphaeria*	0.310***	0.289***	−0.373****	0.362****	−0.345****	−0.101	−0.243**	0.083	−0.139	−0.256**	−0.255**	−0.183*	−0.159*	−0.076	0.096	−0.440****
*Thermococci*	−0.019	0.007	0.188*	−0.115	0.039	0.306***	0.090	−0.138	0.230**	0.091	0.104	0.074	0.226**	0.021	0.215**	0.226**
*Thermoplasmata*	−0.390****	−0.314***	−0.023	−0.403****	0.442****	−0.179*	0.414****	0.160*	0.302***	0.247**	0.293***	0.257**	0.150*	0.308***	−0.219**	0.342****

a Significance: *, *P *≤ 0.05; **, *P *≤ 0.01; ***, *P *≤ 0.001; ****, *P *≤ 0.0001.

**FIG 4:**
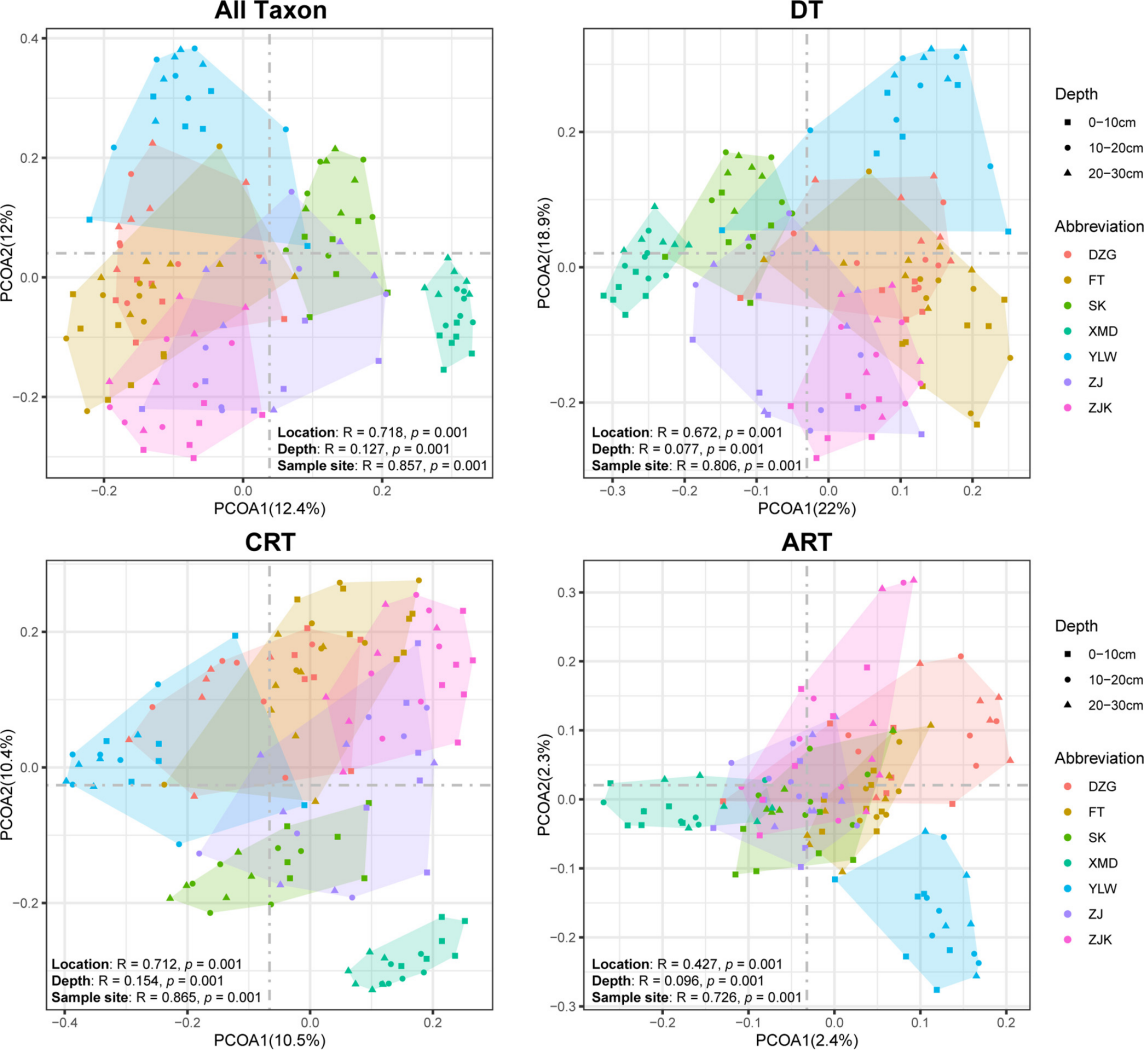
Principal-coordinate analysis (PCoA) derived from the Bray-Curtis dissimilarity matrices displaying the β-diversity of all taxon, dominant taxon (DT), conditionally rare taxon (CRT), and always-rare taxon (ART) communities. Similarity values among the samples of different mangroves (“Location”), depths (“Depth”), and sample sites (“Sample site”) were examined by using the analysis of similarities (ANOSIM) and are shown in the bottom right or left corner of each graph.

Bray-Curtis similarity-based principal-coordinate analysis (PCoA) revealed that, although the percentages on the *x* and *y* axes were very low, the archaeal communities of all taxa, dominant taxa, and conditionally rare taxa were generally clustered according to mangrove location (Fig. 4), which was further supported by permutational multivariate analysis of variance (PERMANOVA) of the archaeal community (*P* = 0.001, Fig. 4). While the percentages on the *x* and *y* axes of the PCoA plot of always-rare taxon communities were much lower than those of the other three plots, the PERMANOVA confirmed the significant difference of always-rare taxon communities in different mangroves (*P* = 0.001, Fig. 4). This indicated significant differences in community composition between mangroves and similar biogeography of the dominant taxon, conditionally rare taxon, and always-rare taxon communities. PERMANOVA also confirmed significant differences in community composition between sample depths (*P* = 0.001) and sample sites (*P* = 0.001). In addition, either for all taxa or for dominant taxa, conditionally rare taxa, and always-rare taxa, sediment depth, location, latitude, longitude, and sample site significantly affected the archaeal communities (*P* ≤ 0.01), and the effects of geographical location and sample site were much stronger than those of other spatial variables (Fig. 4; Fig. S7).

10.1128/mSystems.01381-20.7 FIG S7 Bubble plots showing the influence of spatial variables on the community composition of all taxa (AT), dominant taxa (DT), conditionally rare taxa (CRT), and always-rare taxa (ART) in all and different-layer samples (a) and samples in each mangrove (b). Download FIG S7, JPG file, 1.8 MB. Copyright © 2021 Zhang et al. 2021 Zhang et al. https://creativecommons.org/licenses/by/4.0/ This content is distributed under the terms of the Creative Commons Attribution 4.0 International license.

**FIG 5:**
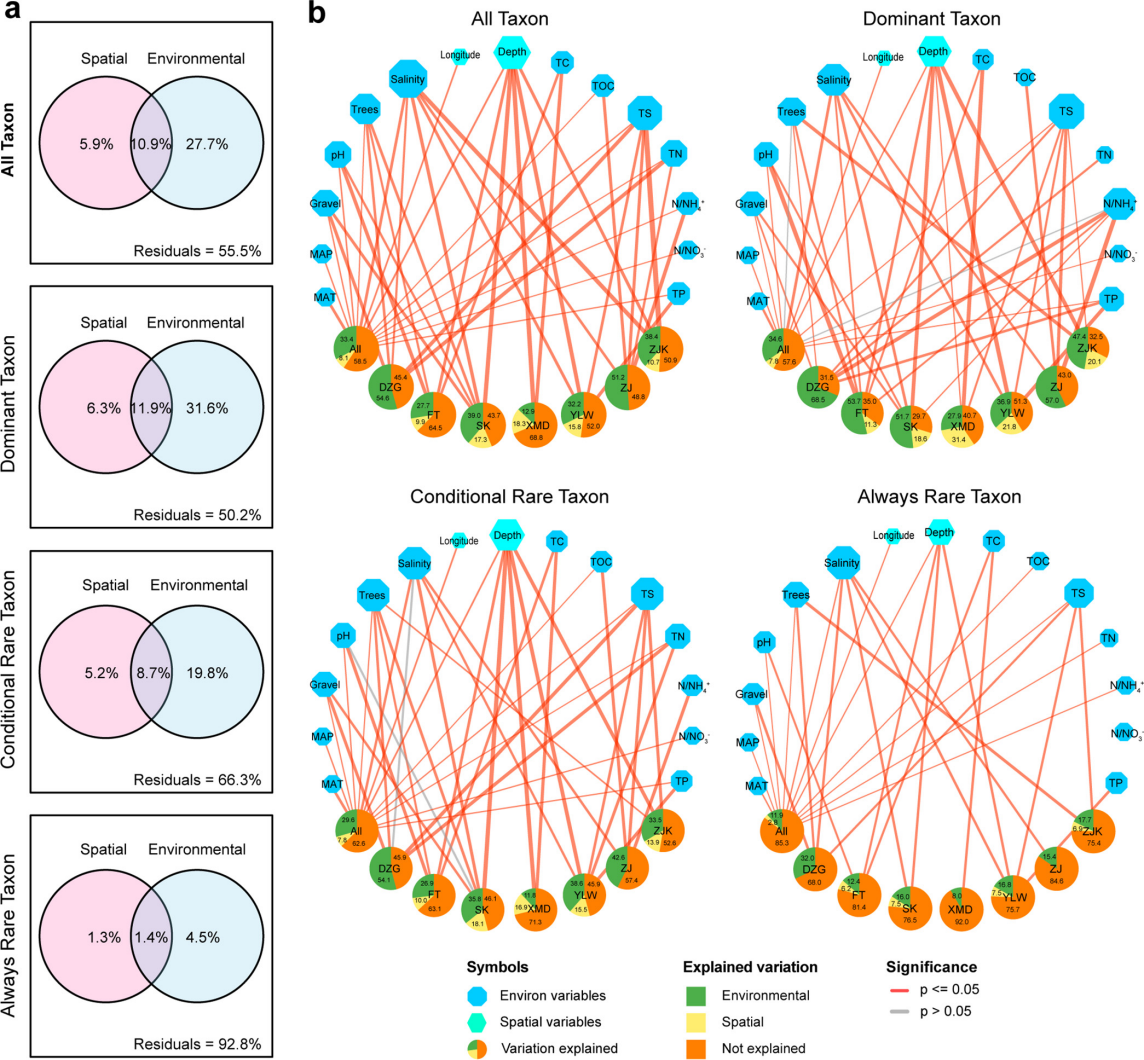
Effect of environmental selection on archaeal community composition. (a) Variation partition analysis based on Bray-Curtis dissimilarity matrices, partitioning the relative contributions of spatial and environmental factors to archaeal community structure. (b) Networks visualizing the results of distance-based redundancy analysis (db-RDA), exhibiting the effects of variables on the archaeal communities of all taxa, dominant taxa, conditionally rare taxa, and always-rare taxa. Upper blue hexagons and octagons, spatial and environmental factors, respectively. Their size represents the number of mangroves influenced by this factor. The bottom pie charts represent the variation of archaeal communities explained by variables in db-RDA models. Positive influences are displayed as red lines, and negative influences are in gray. The variation explained by each variable is displayed by the line width.

The VPA revealed 44.5% variations in the archaeal community explained by spatial and environmental variables. The explained variations of dominant taxon (49.8%) and conditionally rare taxon (33.7%) communities were much higher than that of the always-rare taxon community (7.2%) (Fig. 5a), which was further confirmed by db-RDA (Fig. 5b). All built db-RDA models were statistically validated using ANOVA. For all taxa, a model encompassing 14 variables (longitude, depth, gravel proportion, MAT, MAP, tree number, salinity, pH, TOC, TN, N/NH_4_^+^, N/NO_3_^−^, TP, and TS) was built and explained 41.5% of the archaeal community variation (Fig. 5b). The explanations of models built for seven mangroves ranged from 31.2% (XMD) to 56.3% (SK). For dominant taxa, 12 variables were selected to build an overall community model, which explained slightly more variations (42.4%) than the all-taxon model. The models designed for dominant taxon communities in different mangroves explained 57.0% (ZJ) to 70.3% (SK) of community variation. Further, a model comprising 13 variables was constructed for the conditionally rare taxon community in all samples and explained 37.4% of all variation. The models for the conditionally rare taxon communities in different mangroves explained 28.7% (XMD) to 54.1% (DZG) of community variation. A 12-variable model was constructed for always-rare taxa in all samples, explaining 14.7% of variation; the models built for always-rare taxa in different mangroves explained 8.0% (XMD) to 32.0% (DZG) of community variation (Fig. 5b). Finally, in models built for the all-taxon, dominant taxon, conditionally rare taxon, and always-rare taxon communities, MAT and salinity were the most important factors shaping the archaeal community composition (Fig. 5b).

### Cooccurrence patterns of archaeal community in mangrove sediments.

**FIG 6:**
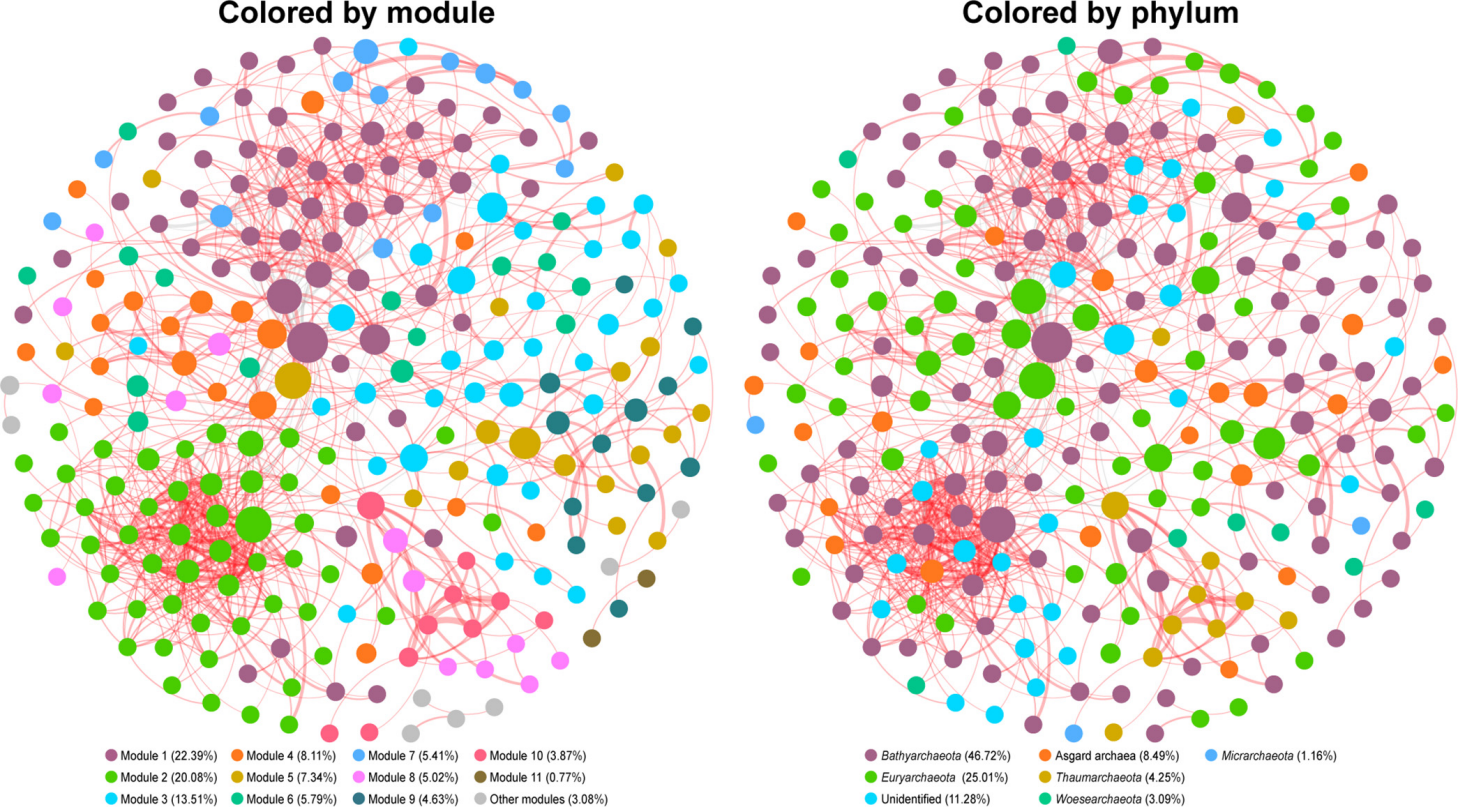
Cooccurrence networks of archaeal communities in all samples. Nodes of networks are colored according to the modularity class and phylum. Node size is proportional to the betweenness centrality of each node, and edge thickness is proportional to the MIC score of each correlation. Positive correlations are displayed as red lines, and negative correlations are in gray.

Based on Spearman’s correlation, a cooccurrence network consisting of 259 nodes (OTUs) and 830 edges was generated for the overall community (Fig. 6). This highly complex network had a diameter of 12, average degree (AD) of 3.21, modularity of 0.65, and average path length (APL) of 4.44. The network consisted of 15 modules, the top 10 of which accounted for 96.14% of the nodes (Fig. 6). The nodes in the network were assigned to six phyla, accounting for 88.72% of all nodes, with 11.28% remaining unidentified at phylum level (Fig. 6). Most edges (94.94%) in the network were positive, indicating that the cooccurring relationships accounted for almost the entire archaeal network (Fig. 6). According to the criteria specified in Materials and Methods, the top 10 OTUs with highest keystoneness scores listed in Data Set S1, sheet 3, were recognized as keystones. All the keystone taxa belonged to module 1 and module 2 (Data Set S1, sheet 3). Seven keystone taxa belonged to *Bathyarchaeota*, one belonged to Asgard archaea (*Lokiarchaeota*), and two were unidentified archaea.

## DISCUSSION

### *Bathyarchaeota*, *Euryarchaeota*, and *Woesearchaeota* are the predominant archaeal groups in mangrove sediments.

Similar to the observations of the current study, *Bathyarchaeota* are the predominant archaeal group also in other mangrove sediments, accounting for up 40.2% of the overall archaeal community (12, 21, 22, 35). However, in some other reports, *Bathyarchaeota* are rare or almost entirely absent, with *Euryarchaeota* and *Thaumarchaeota* dominant (18, 36, 37). In a previous study on the archaeal diversity in DZG, a mangrove site also investigated in the current study, the relative abundance of *Crenarchaeota* was ca. 5 to 10% (36), which is much lower than that noted in the current study (29.6 to 61.7%, mean = 44.5%) and in another study based on the analysis of 16S rRNA gene clones (80.4%) (17). The primer pair 349F/806R used by Li et al. (36) allowed the retrieval of only 11 to 44% of archaeal sequences from each sample, while the Arch519F/Arch915R primers used in the current study retrieved 44.3 to 99.4% of archaeal sequences. The latter therefore provided a more accurate overview of the archaeal community in mangroves. On the other hand, the discrepancy in the reported distribution of *Bathyarchaeota* in different mangroves and samples might be associated with some environmental variables in mangrove wetlands (22). Here, in agreement with previous reports (7, 21, 22), the abundance of *Bathyarchaeota* in subsurface sediment was significantly higher than in surface sediment (see Fig. S3 in the supplemental material) and was positively correlated with sampling depth (Table 1). Further, many *Bathyarchaeota* OTUs were significantly enriched in subsurface sediments compared with surface sediments, while the number of depleted OTUs in subsurface sediments was especially low (Fig. 2a and b). Meanwhile, the Mantel test revealed significant effects of depth on the enriched *Bathyarchaeota* taxa, indicating that depth is a major factor shaping the *Bathyarchaeota* community, in addition to other variables, such as MAT, pH, and salinity (Fig. 2c; Table 1) (22, 38, 39).

*Euryarchaeota* are one of the most predominant archaeal groups, widely distributed in estuarine and mangrove sediments (13, 21, 22, 40). The wide distribution and high abundance of *Euryarchaeota* are a consequence of their pronounced degrading capabilities of diversified substrates and distinct niche adaptations (41–43). Another fascinating observation of the current study was the exceptionally high richness of *Woesearchaeota* (58.9% of all OTUs, 12.8% of sequences). High proportions of *Woesearchaeota* had been recovered from mangrove sediments worldwide, e.g., in China (22, 36, 44) and India (45). This might be attributable to their anaerobic heterotrophic lifestyle and diverse metabolic capabilities, such as the utilization of starch or sugar, acetate fermentation, and metabolism of hydrogen and nitrogen (33, 46), which made them flourish in mangrove sediments, a habitat with nutrient-rich and anaerobic conditions. Unlike *Bathyarchaeota*, the abundance of *Woesearchaeota* in surface sediments was significantly higher than in subsurface sediments and was negatively correlated with sediment depth, indicating that depth significantly impacts the *Woesearchaeota* community, which might be associated with the niche preference of *Woesearchaeota* members (46).

### Homogeneous selection controls archaeal community assembly in mangrove sediments.

The βNTI analysis supported a relatively more crucial role of deterministic processes than that of stochastic processes, either in all (Fig. 3b to d) or in individual (Fig. S4c) mangroves (23, 25, 47). Homogeneous selection is a selection under homogenous abiotic and biotic environmental conditions that leads to increasingly similar structures of various communities (25). Hence, the dominant homogeneous selection observed in the current study might be an outcome of homogenized mangrove habitat (48). However, according to one study of prokaryotic communities in mangrove sediments, heterogeneous selection (βNTI > 2) and stochastic processes (|βNTI| < 2) are dominant in all and individual mangroves, respectively (13). This inconsistency might stem from the notion that only OTUs with an overall abundance above 0.01%, representing the dominant taxa and most conditionally rare taxa, were considered in the previous study, with sediment samples around Spartina alterniflora, an invading marsh grass, included in the analysis (16). The βNTI analysis also revealed an important role of stochastic processes in community assembly, in all and in individual mangroves (Fig. 3d; Fig. S4c). Consistent with the work of Zhang et al. (13), the high fitness of NCM to the archaeal communities in all and individual mangroves (Fig. 3a) indicates the importance of stochastic processes for archaeal community structure (13, 26). Further, regarding the estimated archaeal community immigration rate, the *m* values in each mangrove were higher than that for all mangroves (Fig. 3a), indicating that the dispersal ability of most archaeal taxa within an individual mangrove was higher than that between mangroves. These observations might be attributed to the dispersal limitation and ecological drift that are strongly positively correlated with geographical distance (Fig. S5). Because of the dispersal limitation and ecological drift, longer distance will generate stronger environmental selection, dispersal limitation, and ecological drift and thus results in a distance-decay community similarity (49, 50).

In the current study, dominant taxon communities were mainly driven by dispersal limitation. This result supported prior reports that dominant taxa were mainly limited by dispersion in lakes and reservoirs in China (51), the northwestern Pacific Ocean (52), and agricultural fields in China (48, 53). There are two possible explanations. First, more-abundant species have a greater probability of dispersal than less-abundant species (25, 53). Second, dominant taxa are supposed to have wide niche breadth, and these taxa with wide breadth may be limited by the possibilities to reach multiple locations (dispersal limitation). On the other hand, taxa with narrow niche breadths (rare taxa) may face strong negative environmental selection (52, 54). However, other researchers have also noted stronger limitation of rare taxa than the abundant taxa by dispersal in oil-contaminated soils (55) and subtropical bays (56). These discrepancies may be associated with the difference in the habitat and geographical location (48). The high fraction of “Undominated” processes (41.0%) that contributed to the always-rare taxon community assembly indicates that the always-rare taxon community might be shaped by highly complex assembly mechanisms (48, 56). In summary, we suggest that homogeneous selection strongly shapes the overall and conditionally rare taxon archaeal community assembly and that the stochastic processes play crucial roles in the dominant taxon and always-rare taxon community assembly in mangrove sediments.

### Environmental selection plays a moderate role in shaping archaeal community in mangrove sediments.

In the current study, PCoA revealed distinct community compositions between mangroves; high community similarity within the same mangrove; and similar biogeography of the dominant taxon, conditionally rare taxon, and always-rare taxon archaea in mangrove sediments (Fig. 4). In agreement with these observations, Zhang et al. (13) reported a similar geographical distribution of the prokaryotic community in mangrove sediments, indicating a crucial role of geographical location in shaping archaeal communities in mangrove sediments. The pronounced effects of geographical location are possibly associated with the combined effects of climate, niche conservatism, and rates of dispersal, evolutionary radiation, and extinction in different environments (34, 57). Although the always-rare taxon community did not cluster as well as the all-taxon, dominant taxon, and conditionally rare taxon communities, PERMANOVA supported significant differences between always-rare taxon communities among mangroves (Fig. 4). This was supported by the distance-decay relationship of community similarity and geographical distance (Fig. S5). Similar observations have been made for bacterial communities in coastal Antarctic lakes (58), subtropical bays (56), and microeukaryotic communities in rivers (26).

Consistent with previous studies on fungal, microeukaryotic, or prokaryotic communities in different ecosystems (26, 56, 59, 60), the VPA and db-RDA model built in the current study explained a moderate proportion of variations in all taxon and dominant taxon communities and a relatively low proportion of variations in the conditionally rare taxon and always-rare taxon communities. This indicates a relatively important role of environmental and spatial factors in shaping the all-taxon and dominant taxon communities but a minor role in shaping the conditionally rare taxon and always-rare taxon communities (Fig. 5). Specifically for always-rare taxa, the variables explained only 7.2% (VPA) or 14.7% (db-RDA) of community variation (Fig. 5). As suggested by Chen et al. (26), this observation has several potential explanations. First, some other, more important factors may exist that are not accounted for in the current study. Second, cooccurrence relationships among microbes that significantly affect the community composition cannot be quantified by VPA or db-RDA (61, 62). Third, in some studies, VPA failed to correctly predict the explained community variation and thus should be used together with other approaches, such as NCM (25). Furthermore, for all taxa, always-rare taxa, conditionally rare taxa, and dominant taxa, db-RDA explained a greater proportion of community variation in each mangrove than in all mangroves (Fig. 5), indicating a stronger environmental selection acting on the archaeal community on a small geographical scale rather than a large scale. In conclusion, the above observations reveal similar biogeography of the all-taxon, dominant taxon, conditionally rare taxon, and always-rare taxon communities, a major effect of mangrove location, and a moderate role of environmental selection in driving the archaeal community structure in mangrove sediments.

### Cooccurrence network patterns and keystone taxa in archaeal community in mangrove sediments.

In natural ecosystems, microorganisms preferentially form complex interaction networks rather than thrive in isolation (63). The microbial community composition and dynamics are significantly affected by microbial interactions, and a cooccurrence network constructed using correlation coefficient metrics can provide evidence of interaction between species, such as antagonism or cooperation (61, 62, 64). The mostly positive correlations in the cooccurrence network determined in the current study indicate the possibility that synergy in archaeal communities in mangrove sediments is more frequent than antagonism (64). This phenomenon is quite often observed in natural ecosystems and perhaps is not surprising, because many microbes highly depend on cross-feeding, coaggregation, cocolonization, or niche overlap and construction (65–67). Using anaerobic digesters, Chouari et al. (68) observed a clear cooccurrence of *Crenarchaeota*, *Thermoplasmata*, and methanogens. Consistently, in modules 1 to 5 defined in the current study, close correlations between *Bathyarchaeota* and *Thermoplasmata* or among *Bathyarchaeota*, *Thermoplasmata*, and *Methanomicrobia* were observed (Fig. S8). They indicate that closely cooperative and synergistic interactions might be among these archaea (68). In the current study, seven of the 10 identified keystones were *Bathyarchaeota*. It has been suggested that members of *Bathyarchaeota* can utilize protein, cellulose, short-chain hydrocarbons, and some other recalcitrant organic matters (69). Some *Bathyarchaeota* lineages even have the ability to fix CO_2_ and engage in methane metabolisms (15, 69, 70). Meanwhile, more than 46% of nodes in the overall network represented *Bathyarchaeota*, suggesting that *Bathyarchaeota* might be crucial components maintaining the ecological function and completeness of the archaeal community and playing important roles in the nutrient and biogeochemical cycling in mangrove sediments.

10.1128/mSystems.01381-20.8 FIG S8 Modules 1 to 6 of the overall cooccurrence network (Fig. 6). Nodes in each module are colored by phylum/class. Node size is proportional to the betweenness centrality of each node, and edge thickness is proportional to the MIC score of each correlation. Download FIG S8, JPG file, 1.7 MB. Copyright © 2021 Zhang et al. 2021 Zhang et al. https://creativecommons.org/licenses/by/4.0/ This content is distributed under the terms of the Creative Commons Attribution 4.0 International license.

Although only archaeal communities in mangrove sediment in China were analyzed in the current study, it would be useful to compare the archaeal communities in mangrove sediment and mudflats in different countries. Meanwhile, an annual or seasonal monitoring or average might be more appropriate and precise to demonstrate the structure of the archaeal community in mangroves. However, the above works were not performed here, for several reasons. First, Pan et al. (22) had compared the archaeal communities in mangroves and mudflat sediments in the Futian mangrove in China and observed that the archaeal communities were more similar in the same environments than those in different environments. Second, due to the different PCR primers used in previous studies (7, 71), comparisons between mangroves in China and reported countries were not performed, which may generate bias results due to the different specificities of these primers. Finally, the limited budget of the current study prevented us from the annual and overseas samplings. In the future, temporal variation across seasons and years and spatial variation across continent(s) should be explored to show the overview of archaeal community in mangroves.

### Conclusions.

In conclusion, we found higher archaeal diversity in surface mangrove sediments than in subsurface sediments. *Woesearchaeota* and *Bathyarchaeota* exhibited the highest OTU richness and relative abundance, respectively. In subsurface sediment layers, *Woesearchaeota* were mainly depleted, while *Bathyarchaeota* were largely enriched. Meanwhile, archaeal communities in low-latitude mangroves were significantly more diverse than those in high-latitude mangroves. βNTI analysis and NCM modeling suggested that homogeneous selection strongly shapes the overall and conditionally rare taxon community assembly, whereas stochastic processes play crucial roles in the dominant taxon and always-rare taxon community assembly. PCoA and PERMANOVA revealed similar distribution and biogeography of the all-taxon, dominant taxon, conditionally rare taxon, and always-rare taxon communities. Further, both VPA and db-RDA revealed moderate proportions of community variation explained by spatial and environmental variables. The effects of environmental selection on the always-rare taxon community were weaker than those on dominant taxa and conditionally rare taxa. Mangrove location, MAT, and salinity are the major variables affecting the archaeal community composition. Finally, network analysis revealed comprehensive cooccurrence relationships within an archaeal community, with possible cooperative and synergistic interactions. *Bathyarchaeota* were crucial components of the archaeal community. Overall, these findings provide novel important information on the biogeography, assembly patterns, driving factors, and cooccurrence relationships of archaeal community in mangrove sediments.

## MATERIALS AND METHODS

### Sediment sample collection and variable analysis.

Sediment samples were collected at seven representative mangrove nature reserves in Southeast China in October to November 2019 (see Data Set S1, sheet 1, in the supplemental material). Briefly, six (all except FT) or seven (FT) sampling sites located at the same distance from each other were selected in each mangrove. To compare the vertical profiles of archaeal communities, sediment samples were collected using a stainless-steel sampler and separated into three depth layers (oxic fraction, 0 to 10 cm; anoxic fraction, 10 to 20 cm; and 20- to 30-cm layers) at each sampling sites, according to the criteria of Luis et al. (7). To reduce sampling bias, three replicates were collected at the vertices of an equilateral triangle and mixed together for each site. Overall, 127 sediment samples were collected (Data Set S1, sheet 1).

Data on the spatial and environmental variables are summarized in Data Set S1, sheet 1. The spatial variables included sampling depth, longitude, latitude, and the number of tree species within 5 m of the sampling site. The environmental variables, i.e., tidal height (https://www.cnss.com.cn/tide/), MAT, MAP (obtained from the China Meteorological Administration [http://www.cma.gov.cn]), salinity, pH, gravel proportion, TC, TOC, TN, N/NH_4_^+^, N/NO_3_^−^, TP, and TS, were determined as described by Zhang et al. (60).

### DNA extraction, sequencing, and data processing.

Sediment genomic DNA was extracted from an 0.3-g sample using DNeasy PowerSoil kit (Qiagen, Germany) according to the manufacturer’s instructions. The quantity and quality of extracted DNA were examined using a NanoDrop ND-2000 spectrophotometer (NanoDrop Technologies, USA). The DNA samples were stored at −40°C before amplification by PCR. The hypervariable region 4 (V4) of the archaeal 16S rRNA gene was amplified using the primer pair Arch519F/Arch915R (72, 73). PCR-free libraries were constructed from the 16S rRNA gene amplicons and sequenced using the HiSeq Xten platform with a 450-bp single-end strategy (Illumina, San Diego, CA, USA) at Novogene (Beijing, China).

Raw reads were quality filtered (–q = 20) using Sickle (74). The subsequent replication, singleton and chimera removal, and OTU clustering (at 97% similarity) were performed using USEARCH (75, 76). Taxonomic information for each OTU was assigned by aligning the representative sequences with those deposited in SILVA Nr99 (v138) (77) using BLASTn (78). Bacterial OTUs were removed, and archaeal OTUs were retained. To minimize bias associated with sequencing coverage, the resultant OTU table was rarefied to 19,206 sequences per sample for downstream analyses. The relative abundance of individual taxa was calculated by comparing the read number of each taxon with the total read number in that sample. In the current study, all archaeal OTUs (all taxa) were classified into three categories: dominant taxa, OTUs with the relative abundance of ≥0.01% in all samples or ≥1% in some samples; always-rare taxa, OTUs with the abundance of <0.01% in all samples; and conditionally rare taxa, with the abundance of <1% in all samples and <0.01% in some samples (26, 28).

### Statistical analysis.

**(i) Different taxa in subsurface sediments.** Enriched and depleted OTUs in subsurface-layer samples (10 to 30 cm) compared with surface-layer samples (0 to 10 cm) were analyzed using metagenomeSeq package (79). Using a log transformation [log_2_(yij + 1)] followed by correction for zero-inflated log-normal model, metagenomeSeq was specifically designed for the differential abundance analysis of amplicon sequencing and had a substantially best performance for microbial marker-gene surveys (79, 80). According to the work of Paulson et al. (81), a zero-inflated log-normal model implemented in the fitFeatureModel function in the metagenomeSeq package was used for the differential abundance testing for OTUs in surface and subsurface sediments. The effects of spatial and environmental variables on the enriched and depleted OTUs in subsurface sediments were explored using the Mantel test in vegan package (82), to determine Spearman’s correlation coefficient between the Bray-Curtis distance of enriched or depleted OTUs and Euclidean distance of spatial and environmental variables based on 9,999 permutations.

**(ii) α- and *β*-diversity analysis.** α-Diversity indices, including OTU richness, Shannon-Wiener, Chao1, and evenness indices, were calculated using the vegan package (82). ANOVA followed by Tukey’s honestly significant difference method was used to explore the variations of α-diversity and relative abundances of major phyla across different depths (83). Pearson’s correlation coefficients and *P* values were calculated to explore the associations between α-diversity, main archaeal phyla and classes, and environmental features. The distance matrix of the archaeal community (Hellinger transformation of the OTU abundance data) was constructed by calculating dissimilarity using the Bray-Curtis method (82). In the current study, PCoA was employed using the Bray-Curtis similarity matrices to visualize shifts in different archaeal community compositions. Significant differences (*P* < 0.05) among groups were evaluated using the analysis of similarities (ANOSIM).

**(iii) Archaeal community assembly patterns.** NCM was used to determine the potential roles of stochastic processes in archaeal community assembly by predicting the relationship between OTU detection frequency and the OTU’s relative abundance in the whole community (26, 84, 85). The model used here is derived from the neutral theory (25, 26). The model predicts that taxa that are abundant in the community will be widespread, since they are more likely to disperse by chance among different sampling sites, while rare taxa are more likely to be lost due to the ecological drift (26). In this model, parameter *m* represents the immigration rate, and *R*^2^ represents the overall fit to the neutral model. The sncm.fit_function.r script written by Burns et al. (86) was used to evaluate the NCM fit. Further, Pearson’s correlation coefficients between Bray-Curtis similarity of the archaeal community and geographic distance of the samples were calculated to determine the spatial predictor of archaeal community composition. The geographical distance (in km) between samples, i.e., a straight-line distance between the sampling points, was calculated using the package geosphere (87) based on the longitude and latitude coordinates of each sampling site.

To quantify the relative importance of stochastic and deterministic processes that drive the archaeal community assembly, the null model analysis was performed using Rscript bNTI_Local_Machine.r written by Stegen et al. (23), based on the phylogenetic distance and OTU abundance. This method can be used to detect community assembly mechanisms by estimating the standard deviation of the observed ecological patterns compared to the randomly shuffled ecological patterns produced by null models (23, 47). If the observed ecological patterns are not statistically different from null expectations, the community dynamics are largely considered stochastic; otherwise, they are regarded as deterministic (53). Briefly, βNTI is the number of standard deviations of the beta mean nearest taxon distance (βMNTD) from the mean of the null distribution (23). The RC_bray_ value was used to further partition pairwise comparisons that were assigned to stochastic processes (23, 25, 53). The βNTI values between −2 and 2 indicate dominance of the stochastic processes, whereas βNTI values smaller than −2 or larger than 2 indicate that deterministic processes (i.e., homogeneous selection and heterogeneous selection) play a more important role in community assembly than stochastic processes (13, 23, 25, 53). For |βNTI| < 2, RC_bray_ < −0.95 and RC_bray_ > 0.95 indicate relative dominant influence of homogenizing dispersal and dispersal limitation, respectively, and |RC_bray_| < 0.95 indicates a crucial role of “Undominated” assembly, including weak selection, weak dispersal, diversification, and/or drift (25, 53, 88). The major factors that influence the assembly of dominant taxa, conditionally rare taxa, and always-rare taxa were explored separately. Pearson’s correlation coefficients and *P* values were calculated to explore the associations between βNTI values and changes in environmental variables in different samples.

**(iv) Archaeal community-driving factors.** VPA based on db-RDA was performed to determine the relative proportions of community variation that could be explained by spatial (latitude, longitude, and sediment depth) and environmental (MAT, MAP, salinity, pH, gravel proportion, TC, TOC, TN, N/NH_4_^+^, N/NO_3_^−^, TP, and TS) variables combined. To assess the relative impact of each variable on archaeal community structure, db-RDA with Bray-Curtis dissimilarity analysis was performed, exploring the community variations explained by spatial and environmental variables (89). Using vif.cca and ordiR2step function in the vegan package (82), the method described by Zhang et al. (60) was used to select parameters for the db-RDA model. Briefly, the final db-RDA model was comprised of variables forward-selected by ordiR2step with variance inflation factor (VIF) values below 10. The significance of the final model, axes, and terms was tested using the ANOVA command.

**(v) Network patterns and keystone taxa of archaeal communities.** Network analysis reveals the interactions within an archaeal community and keystone taxa within cooccurrence networks that are crucial for the composition and assembly of the community (64). The cooccurrence patterns in archaeal communities were determined by performing network analysis using the maximal information coefficient (MIC) scores (90). The MIC is a useful score that reflects the strength of linear and nonlinear associations among variables (90). To reduce the influence of false positives caused by OTU sparsity, OTUs that were present in at least half of the samples (i.e., 50% OTU sparsity) were included (91), resulting in 534 OTUs. The pairwise MIC associations were analyzed in MICtools with default parameters (92), and the *P* value was adjusted using false-discovery rate correction. Of the correlations, only those with adjusted *P* values below 0.001 were considered statically robust. Since MIC roughly equals the coefficient of determination *R*^2^ between two variables (85, 90, 92), a MIC cutoff of 0.5, which represented a relatively strong coefficient (|*R*| > 0.7), was selected to construct the network (93). Meanwhile, the MIC cutoff also generated a moderate network size, 259 nodes and 830 edges. Cooccurrence networks were built using the igraph package (94); calculation of network topological properties and network visualization were performed using the interactive platform Gephi v0.92 (95). In the network, a degree represents the number of edges connected to a node. Closeness centrality (CC) is based on the average shortest paths and thus reflects the central importance of a node in disseminating information (63). Betweenness centrality (BC) reveals the role of a node as a bridge between network components. Keystone species are defined as highly connected species that have a disproportionately large effect on the complexity of a community relative to their abundance (64, 96). Keystone taxa were OTUs with the highest degree and CC scores and the lowest BC score (64). Putative keystone OTUs in the networks were identified by calculating a keystoneness score by the average of degree, 1-BC score, and CC score after Mix-Max scaling (64, 97, 98).

### Data availability.

All raw sequences from the current study have been deposited in NODE (http://www.biosino.org/node) under the accession number OEP001412.
