# Supplementary figures and images for: Biogeography, Assembly Patterns, Driving Factors, and Interactions of Archaeal Community in Mangrove Sediments
Source: mSystems. 2021 Jun 15;6(3):e01381-20. doi: 10.1128/mSystems.01381-20 (PMC8269266; doi:10.1128/mSystems.01381-20)

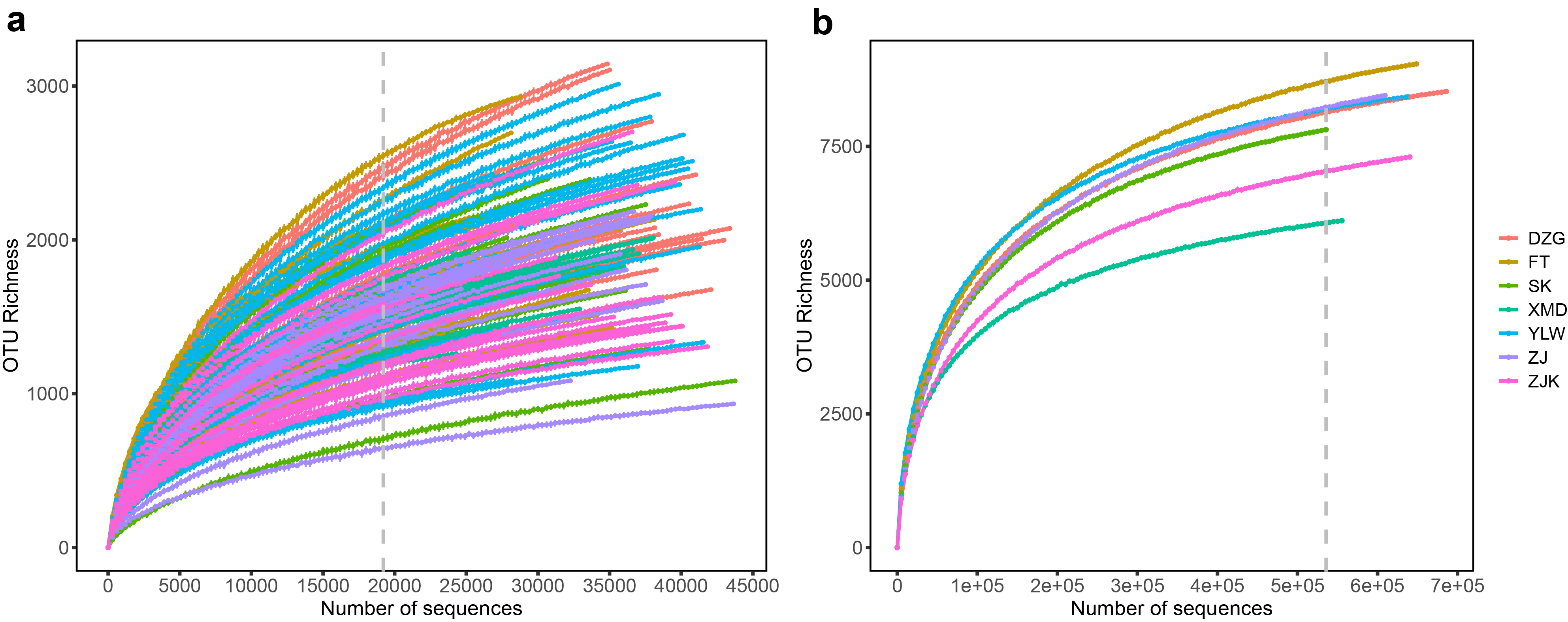

Supplement: FIG S1 [file msystems.01381-20-sf001.jpg]

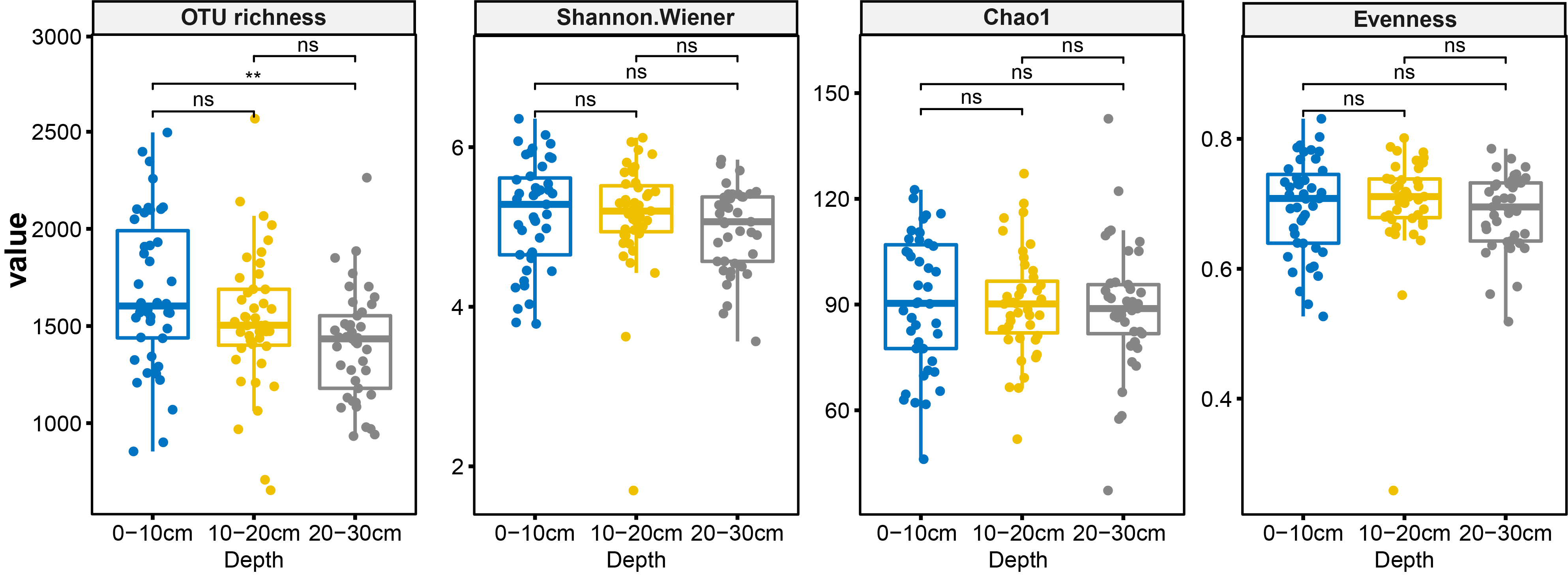

Supplement: FIG S2 [file msystems.01381-20-sf002.jpg]

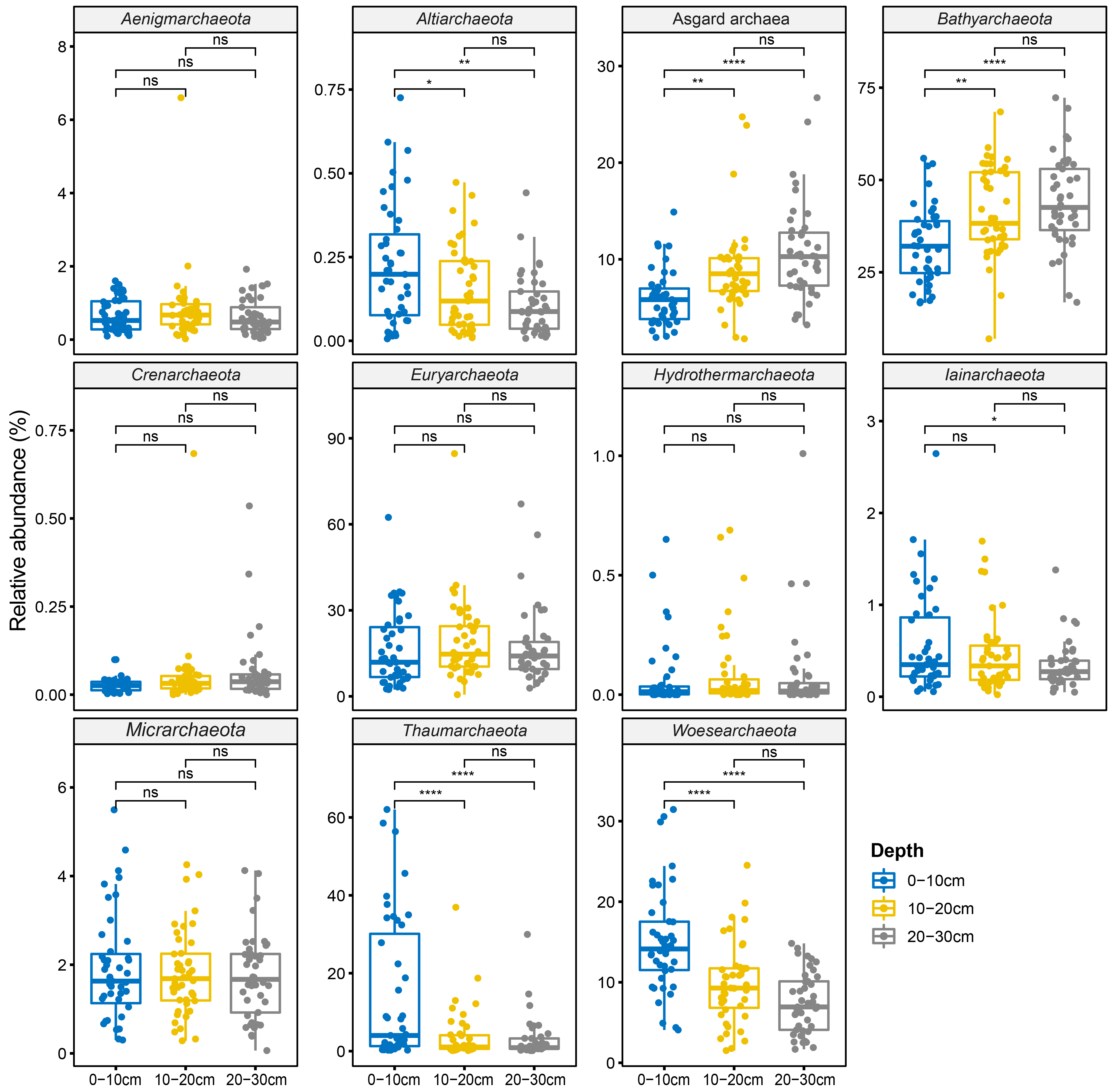

Supplement: FIG S3 [file msystems.01381-20-sf003.jpg]

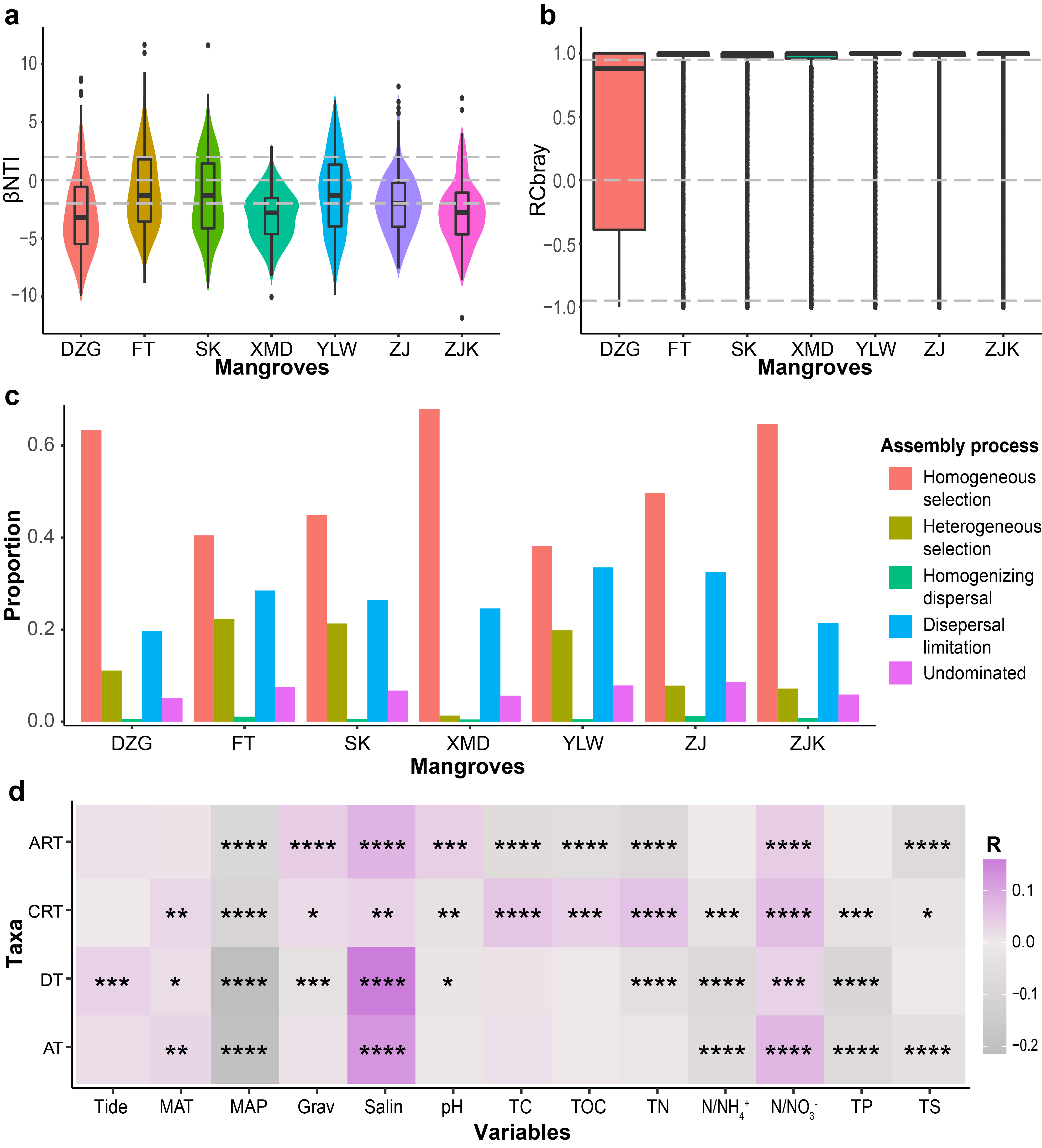

Supplement: FIG S4 [file msystems.01381-20-sf004.jpg]

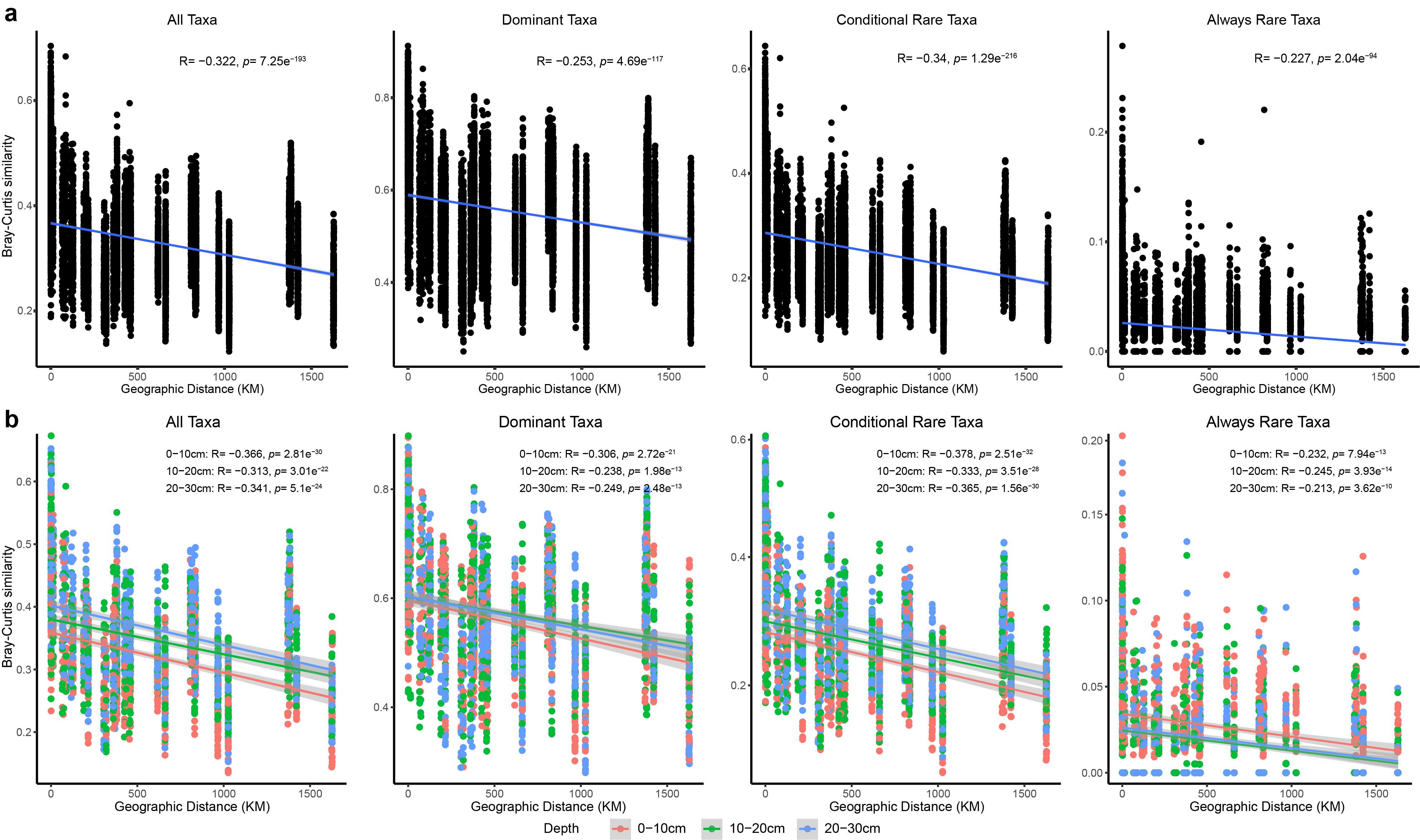

Supplement: FIG S5 [file msystems.01381-20-sf005.jpg]

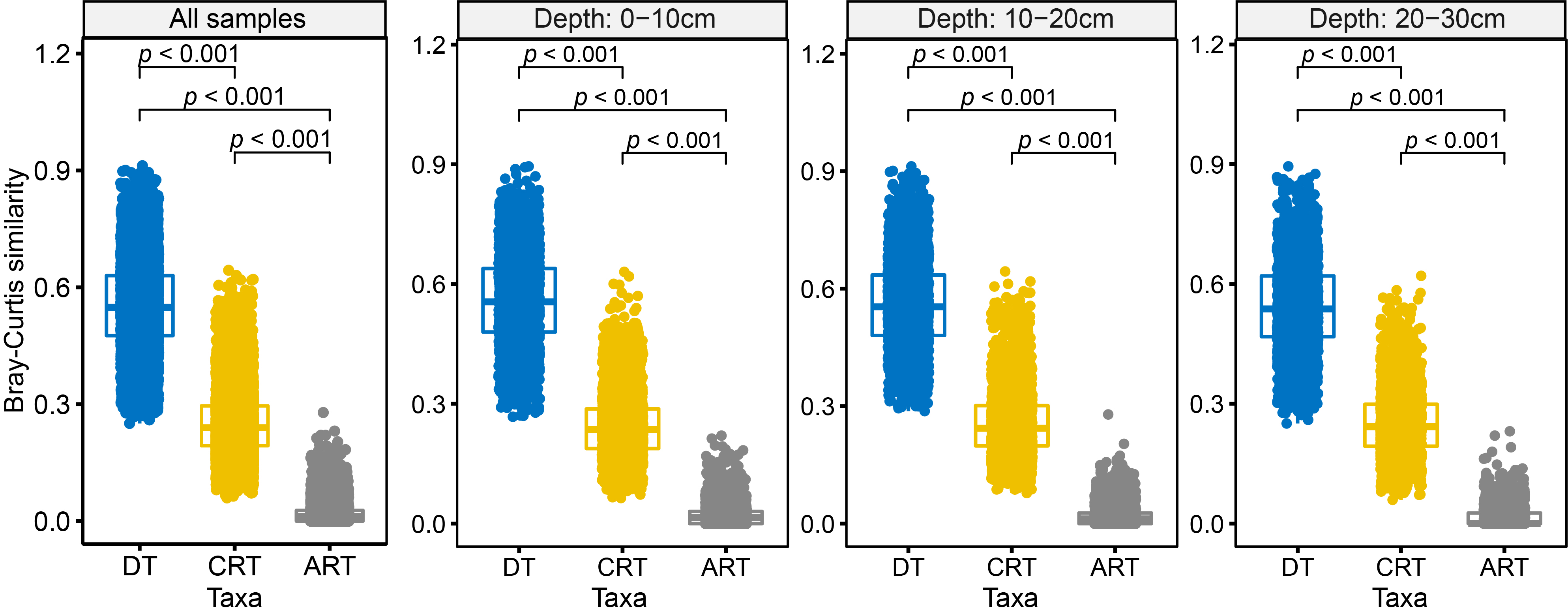

Supplement: FIG S6 [file msystems.01381-20-sf006.jpg]

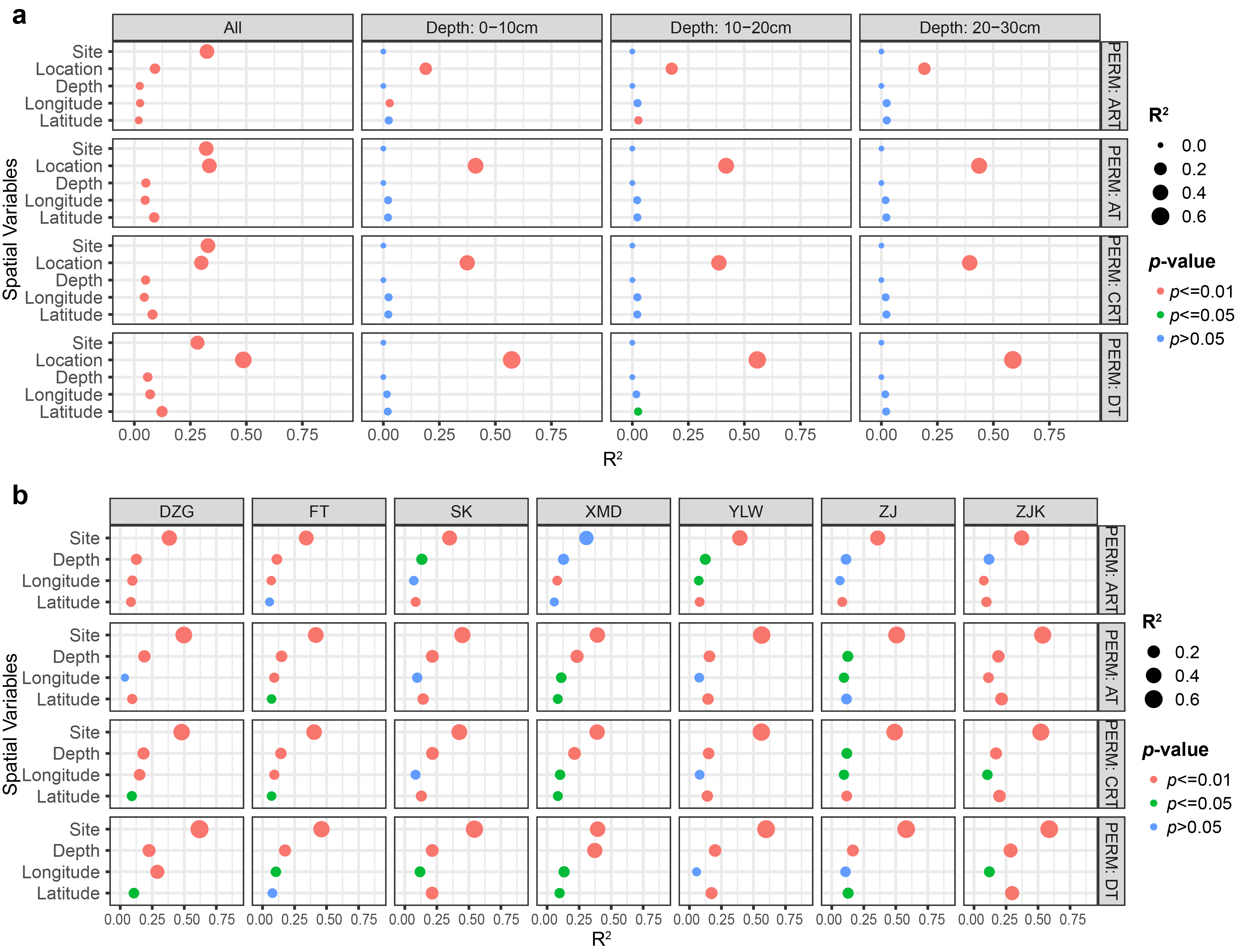

Supplement: FIG S7 [file msystems.01381-20-sf007.jpg]

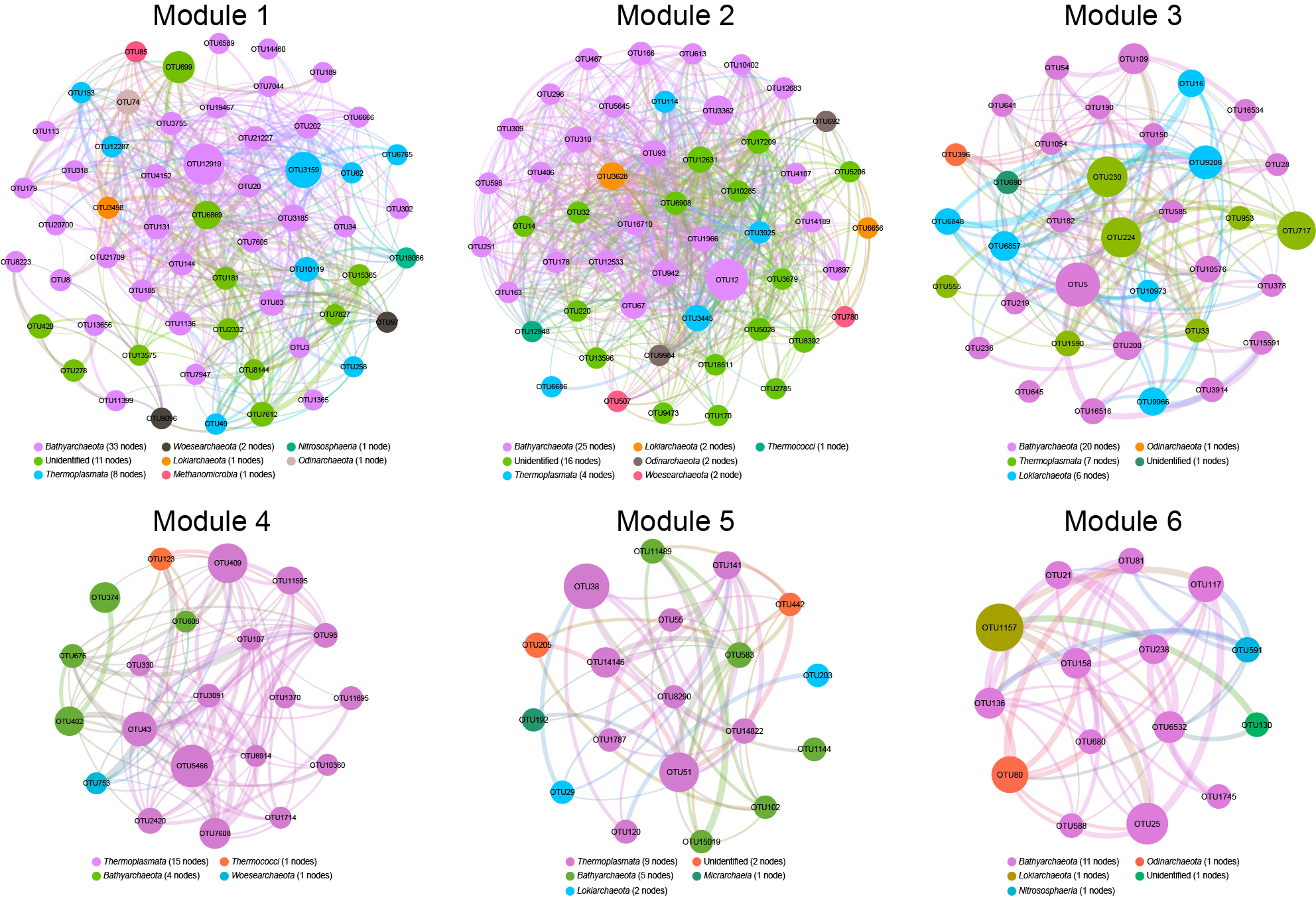

Supplement: FIG S8 [file msystems.01381-20-sf008.jpg]
